# Lack of frequency-tagged magnetic responses suggests statistical regularities remain undetected during NREM sleep

**DOI:** 10.1038/s41598-018-30105-5

**Published:** 2018-08-06

**Authors:** Juliane Farthouat, Anne Atas, Vincent Wens, Xavier De Tiege, Philippe Peigneux

**Affiliations:** 1 UR2NF - Neuropsychology and Functional Neuroimaging Research Unit at CRCN, Center for Research in Cognition and Neurosciences, Université libre de Bruxelles, Brussels, Belgium; 2 CO3 - Consciousness, Cognition, and Computation Group at CRCN, Center for Research in Cognition and Neurosciences, Université libre de Bruxelles, Brussels, Belgium; 3 LCFC - Laboratoire de Cartographie Fonctionnelle du Cerveau, Université libre de Bruxelles, Brussels, Belgium; 4 UNI - ULB Neurosciences Institute, Université libre de Bruxelles, Brussels, Belgium

## Abstract

Hypnopedia, or the capacity to learn during sleep, is debatable. *De novo a*cquisition of reflex stimulus-response associations was shown possible both in man and animal. Whether sleep allows more sophisticated forms of learning remains unclear. We recorded during diurnal Non-Rapid Eye Movement (NREM) sleep auditory magnetoencephalographic (MEG) frequency-tagged responses mirroring ongoing statistical learning. While in NREM sleep, participants were exposed at non-awakenings thresholds to fast auditory streams of pure tones, either randomly organized or structured in such a way that the stream statistically segmented in sets of 3 elements (tritones). During NREM sleep, only tone-related frequency-tagged MEG responses were observed, evidencing successful perception of individual tones. No participant showed tritone-related frequency-tagged responses, suggesting lack of segmentation. In the ensuing wake period however, all participants exhibited robust tritone-related responses during exposure to statistical (but not random) streams. Our data suggest that associations embedded in statistical regularities remain undetected during NREM sleep, although implicitly learned during subsequent wakefulness. These results suggest intrinsic limitations in *de novo* learning during NREM sleep that might confine the NREM sleeping brain’s learning capabilities to simple, elementary associations. It remains to be ascertained whether it similarly applies to REM sleep.

## Introduction

A popular idea in the ‘50 s, hypnopedia was rapidly questioned by controlled experiments concluding to a lack of learning during sleep^1–3^. Successful recall of information presented during sleep was associated with increased electroencephalographic (EEG) alpha activity shortly after item presentation^1,4,5^, suggesting that delayed retrieval in earlier experiments was due to increased alertness and transition toward wakefulness after stimulus presentation.Lack of learning during sleep is compatible with a well-documented decreased responsiveness to external stimulation. Notwithstanding, residual sensory perception during sleep exists in olfactory^6^, tactile^7–9^, visual^8^ and auditory^8,10–16^ modalities. Short-latency evoked related potentials (ERPs) components (i.e., mirroring primary cortical activity) especially bear similarities with those recorded during wakefulness, whereas middle and long-latency components reflecting higher-level processes are more often distorted^8^. Furthermore, experimental data indicate that higher cognitive processes are not entirely inefficient during sleep. Residual cognitive abilities have been evidenced in the domains of semantic processing^11,17^, working memory^18^, detection of irregularities^19^ and extraction of information for motor response preparation^20,21^. Targeted memory reactivation (TMR) during sleep was also found to promote memory consolidation processes. Auditory or olfactory presentation during post-training sleep of items previously associated with the learned material improved memory retention above expected sleep-related consolidation benefits, thus suggesting interactions between memory content and sensory stimulation during sleep (see^22–24^ for reviews).

Recently, conditioning odour-related respiratory reflex to tones was successfully established during sleep in young adults, and the association preserved at wake^25,26^. These results provided a first rigorous experimental demonstration of the possibility to learn simple associations during human sleep, like in rodents^27^. Additionally, perceptual auditory learning that transferred to wakefulness was found efficient during REM sleep and light NREM2 sleep, but detrimental in deep NREM3 sleep^28^. Whether more sophisticated forms of learning are possible during sleep remains an open question. In infants, prior studies evidenced vowels detection training in seemingly sleeping newborns^29^, statistical language learning in newborns exhibiting active sleep^30^, and anticipatory auditory responses in 3-month-old infants in behavioural quiet sleep^31^. However, lack of polysomnographic measurements could not preclude the possibility that learning occurred during arousal, wake-like periods. Furthermore, the infant’s sleep is immature and disorganised^32^, and exhibit noticeable functional differences with adult sleep^33^. Altogether, available evidence suggests that acquisition of novel information during adult human sleep might restrict to elementary discriminative or conditioning processes^34,35^.

Statistical learning is the detection and retention of statistical regularities, such as the segmentation of continuous streams of syllables based on transitional probabilities between successive elements. This ability is present at wake in adults and children^36,37^, and in seemingly sleeping newborns^30^. Detecting statistical information within a flow of syllables is thought one of the key mechanisms subtending first-language acquisition in children^37^. It occurs incidentally, i.e. without explicit instructions about the detection of patterns but can be impaired when attention is diverted toward a concurrent task^38^. This makes statistical learning an interesting candidate to investigate learning abilities during sleep. Indeed, demonstrating that sleeping humans can detect statistical regularities would suggest the possibility to learn novel stimulus-stimulus associations during sleep. In this framework, we aimed at determining whether young adults can detect auditory statistical regularities during NREM sleep, and whether such learning can transfer during a subsequent wakefulness period. At the behavioural level, statistical learning is usually assessed using two-alternative forced choice (2AFC) tests^36^. That is, after passive exposure to the regularities embedded in the material, participants must discriminate between items constructed using high versus low (or null) transitional probabilities. Statistical learning is evidenced when participants exhibit a trend to select high transitional probability items, even if unaware of the presence of the regularities. However, behavioural tests are often lacking sensitivity^39–42^. Electrophysiological^39^ (EEG) and magnetoencephalographic^40^ (MEG) frequency-tagged responses have been shown to mirror the ongoing detection of statistical regularities. The principle of frequency tagging is that if a stimulus is systematically presented in a periodic, regular manner, then the neural population coding for that stimulus will be entrained to the same period^43^. The frequency of stimulus occurrence thus provides a target frequency tag to identify the associated brain response, which emerges as a peak in the power spectrum of the brain signal with a strong signal-to-noise ratio (SNR) as compared to neighbouring frequencies. This approach allows investigating stimulus-related neural responses at the individual level^44^, a particularly interesting feature for sleep learning studies^3^. Frequency-tagged responses also overcome the problems of overlapping ERPs in fast continuous streams of stimuli^39,45,46^, and possibly the overlap between auditory responses and sleep-specific oscillations (such as slow waves, K-complexes or spindles) during sleep.

**Figure 1:**
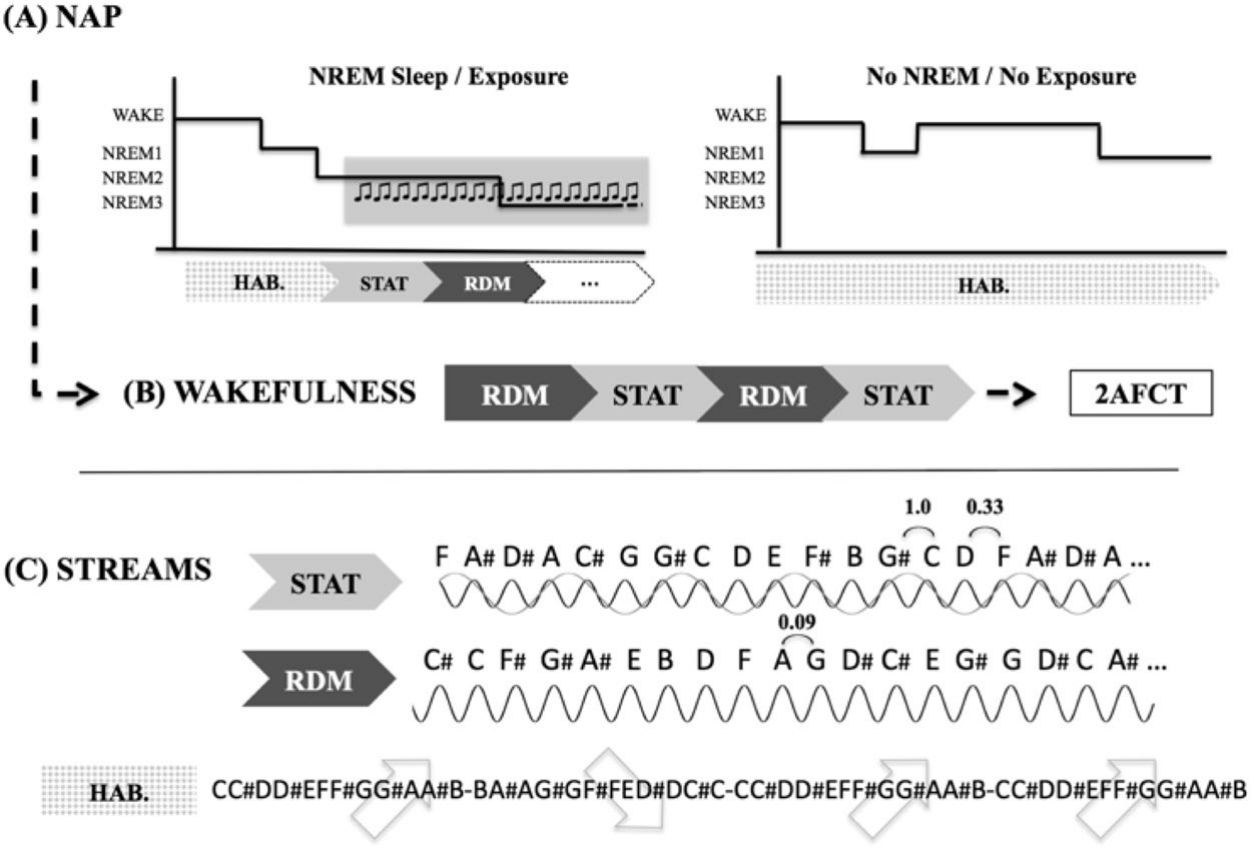
(**A**) In the Sleep (nap) MEG session, participants are delivered habituation auditory streams (HAB; regularly ascending/descending half-tone scales) while falling asleep. When stable NREM2/NREM3 sleep stages are observed, the experimenter delivers statistical (STAT) and random (RDM) 5-minutes auditory streams (counterbalanced). Participants who do not reach NREM sleep or cannot be delivered STAT streams are assigned to the *No NREM*/*No Exposure group* (N = 10); others are assigned to the *NREM Sleep*/*Exposure group* (N = 11). (**B**) In the subsequent MEG session at Wake, both groups are delivered 5-minutes STAT and RDM streams (2 times each, counterbalanced). Outside of the scanner, they are then informed of the presence of regularities, and administered a two alternative forced choice (2AFC) test between trained and never seen tritones. (**C**) Auditory streams. STAT streams feature non-melodious successions of 12 pure tones that can be statistically grouped into four randomly alternating tritones (G#CD, AC#G, FA#D# and EF#B in musical notation, A = 440 Hz). Transitional succession probability between two tones within a tritone is 1.0, and transitional probability between two tritones is 0.33, creating statistical grouping regularities. In RDM streams, the same 12 tones are randomly presented (transitional probability between tones = 0.09). Habituation (HAB) streams are constructed with the same 12 tones. Tones are delivered at a 5.505 Hz presentation rate, and tritone presentation rate is 1/3 of the tone = 1.835 Hz (see Methods).

In this study, we recorded magnetoencephalographic (MEG) frequency-tagged brain responses previously shown to mirror the covert acquisition of statistical regularities at wake^40^ to test whether young adults can learn high-order auditory regularities during sleep, and transfer this information to subsequent wakefulness. All participants gave a written, signed informed consent prior to this experiment performed in accordance with the relevant guidelines and regulations, and approved by the ULB-Erame Ethics committee. Twenty-one young adults had a 90-minutes afternoon nap opportunity in the MEG scanner. While in unequivocal Non-Rapid Eye Movement sleep stages NREM2 or NREM3 sleep^47^, they were exposed at non-awakening thresholds to alternating 5-minutes blocks of statistical (STAT) pure tone streams, in which tones statistically grouped as triplets, and random (RDM) streams, in which the succession of tones was random (see Fig. 1 and Methods).

Stimulus (tone) presentation rate was 5.505 Hz. If statistical regularities (tritones) embedded in the auditory stream are detected during sleep, a brain response will emerge at the frequency of occurrence of each tritone onset (1.835 Hz) in the STAT stream, alongside tone frequency-tagged auditory responses (5.505 Hz) present both in STAT and RDM conditions that reflect the mere detection of auditory stimulations. Half of the participants (n = 11) reached sufficient levels of NREM sleep to receive at least 5 minutes of STAT auditory stimulation. In a second step, all participants (previously exposed vs. not) were scanned again in the wakefulness state while exposed to the STAT and RDM auditory streams. At this stage, frequency-tagged MEG analyses aimed at testing whether participants previously exposed to the STAT stream during sleep would exhibit better learning (as compared to those not exposed), i.e. would detect faster the tritone structure in the material, which would evidence a transfer of the learned information from sleep to wakefulness.

## Results

### Fatigue and sleepiness

Subjective fatigue and sleepiness scores before and after the nap opportunity phase are reported Table 1. A repeated measure ANOVA conducted on subjective sleepiness scores with the within-subject factor Moment (pre-Nap vs. post-Nap) and the between-subjects factor Group (NREM/Exposure vs. No NREM/No Exposure) failed to disclose significant main or interaction effects (*ps* > 0.09), suggesting similar sleepiness levels before and after the nap in both groups. A similar repeated measure ANOVA conducted on subjective fatigue scores disclosed a main Moment effect (F(1,16) = 5.0, p = 0.04), with higher subjective fatigue after than before the nap episode. Main Group and Moment by Group interaction effects were non significant (*ps* > 0.5).

**Table 1:** Subjective fatigue and sleepiness mean scores ( +/− sd) before and after the 90-minutes nap opportunity.

	Moment	VAS Fatigue	VAS somnolence	KSS
NREM/Exposure	before	5.2 +/− 2.1	3.6 +/− 2.6	3.2 +/− 1.1
	after	7.0 +/− 1.7	2.4 +/− 1.5	2.6 +/− 1.4
No NREM/No Exposure	before	6.0 +/− 2.3	3.2 +/− 1.8	4.2 +/− 1.7
	after	7.2 +/− 1.8	2.8 +/− 2.1	3.3 +/− 1.6

Note: VAS: visual analog scale; KSS = Karolinska Sleeping Scale^96^.

### Frequency-tagged responses to STAT and RAND streams during NREM sleep

Eleven participants were included in the NREM/Exposure condition as they were exposed to at least 5 minutes of the STAT auditory stream during NREM2 and/or NREM3 sleep stages (2 participants were exposed only to the STAT, but not to the RAND stream). On average, participants were exposed to STAT streams during 4.1 +/− 2.3 minutes in stage NREM2 and 5.9 +/− 4.7 min in stage NREM3 (Wilcoxon signed-rank test, W = 48.0, p = 0.40), and to RDM streams during 3.0 +/− 3.8 minutes in stage NREM2 and 7.2 +/− 5.7 min in stage NREM3 (W = 33.5, p = 0.073). Global sleep parameters derived from polysomnographic recordings are reported Table 2. For the NREM/Exposure condition, individual hypnograms illustrating the timing of exposure to STAT and RDM streams are reported in Supplementary Material Fig. S1, and individual duration of exposure per sleep stage and stream is reported in Supplementary Material Table T1. For the No NREM/No Exposure group, individual duration of (insufficient) exposure to RAND and STAT streams is reported in Supplementary Material Table T2.

**Table 2:** Sleep parameters in the NREM/Exposure group during the 90-minutes nap opportunity

	TAT	TST	Wake	NREM1	NREM2	NREM3	REM
[min]	79.2 +/− 11.3	55.6 +/− 20.6	23.4 +/− 17.8	9.0 +/− 5.0	25.9 +/− 11.5	16.5 +/− 11.8	4.2 +/− 7.9
[%TST]	—	—	—	18.4 +/− 11.1	48.8 +/− 16.0	27.0 +/− 20.0	5.7 +/− 10.2

Note. TAT: total acquisition time. TST: total sleep time. Reported values are mean minutes or percentage of TST ( +/− standard deviations) across participants.

**Figure 2:**
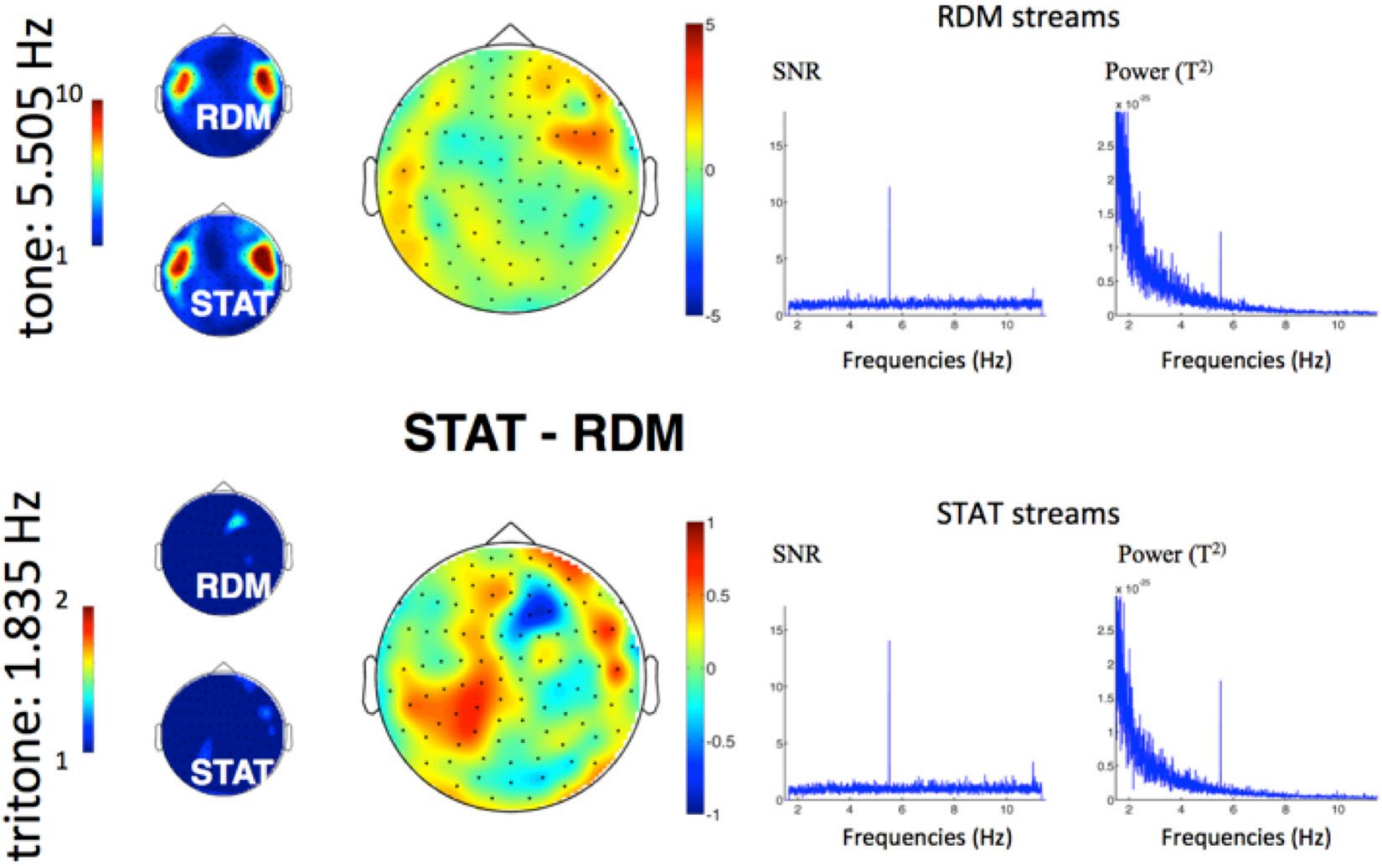
MEG frequency-tagged responses during NREM sleep for tones (5.505 Hz; top) and tritones (1.835 Hz; bottom) in STAT and RDM streams, averaged across participants (n = 9). Left column: Topographies of tone-related (up) and tritone-related (bottom) frequency-tagged responses. No significant tritone-frequency tagged response is evidenced either for STAT or RDM streams. Middle column: topographies of (non-significant) differences between STAT and RDM tone- (top) and tritone- (bottom) related frequency-tagged responses (note that colour value scales range [−5 + 5] for ones, and [−1 + 1] for tritones). Right column: SNR and power spectra (combined gradiometer 1332 + 1333 over temporal sensors) averaged for RDM (top) and STAT (bottom) streams with a strong 5.505 Hz peak at the tone-related frequency.

First, we assessed global/overall segmentation during the sleep exposure. To do so, power was computed on averaged continuous 5-min streams. Topographies of tone- and tritone-related responses as well as SNR and power spectra are illustrated in Fig. 2. Individual topographies are available as Supplementary Material Fig. S2. Note that comparisons between STAT and RDM streams were conducted on 9 participants, excluding the two participants exposed to STAT streams only during sleep. Neural responses tagged at the tone presentation rate during sleep were found in all participants but one (who was excluded from future analyses), reflecting auditory stimulation processing. Tone-SNR was particularly strong (SNR > 10) in bilateral temporal sensors, and did not differ between STAT and RDM sequences (cluster-based permutation test; p > 0.1; Fig. 2, top panel). At the tritone presentation rate however, no frequency-tagged response was evidenced either for STAT or RDM streams, and tritone-related SNR did not differ between streams (cluster-based permutation test; p > 0.09; Fig. 2, bottom panel). Lack of between-group differences at the tritone level was substantiated by a Bayesian paired sample t-test in favour of the null hypothesis (BF10 = 0.33). At the tone level, the Bayesian factor for between-group differences was inconclusive (BF10 = 0.43), although far from the BF value > 3 needed to support the alternative hypothesis of between-group differences. Similar sensor space analyses performed at the first harmonic of each frequency of interest (FOI) disclosed similar results (see Supplementary Material Fig. S3). SNR and power spectra (Fig. 2, right column) disclosed an emergent peak at the tone-related frequency in both RDM and STAT streams. To the contrary, no SNR or power spectra peaks emerged at the tritone-related frequency or its first harmonics. These results evidence the successful auditory perception of individual tones during sleep, but no learning of the auditory regularities.

**Figure 3:**
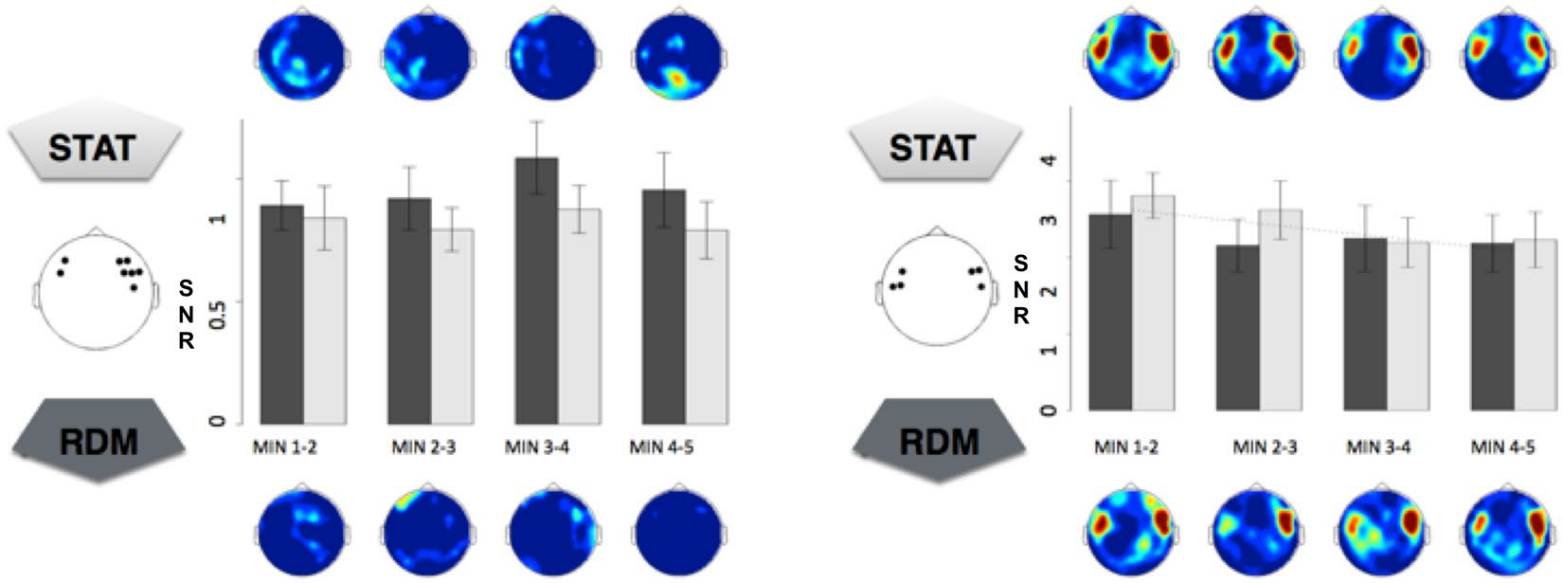
Temporal dynamics of tritone-SNR (left) and tone-SNR (right) during the first 5-minutes exposure to STAT and RDM streams during sleep. Topographies related to STAT and RDM streams correspond to the upper and lower row respectively. Averaged SNR within sensors of interest is represented in light grey bars for STAT streams and dark grey bars for RDM streams.

Second, we examined using linear mixed models the temporal evolution of tone- and tritone-related responses during the first 5 minutes of continuous STAT and RDM streams. The time course of the tritone- and the tone-related frequency-tagged responses is illustrated in Fig. 3. For tritone-related responses, random effects included the intercepts. The model that best accounted for tritone-related SNR included the predictor STREAM, i.e., tritone-related SNR ~ STREAM (notation A ~ B indicates A depends on B), but the effect was not significant (p > 0.1). For tone-related responses, random effects included the intercepts and the slopes for the MINUTES predictor. The most appropriate model was the one including the continuous MINUTES predictor (i.e., SNR ~ MINUTES), but the effect was also not significant (p > 0.2).

### Frequency-tagged responses to STAT and RAND streams during subsequent Wake

One could argue that the subset of participants stimulated during sleep merely had poor learning abilities. Also, it cannot be excluded that even in the absence of overt brain responses during sleep, exposure to auditory regularities during sleep would facilitate subsequent learning. To address these issues, all participants went back in the MEG scanner 30 minutes after the end of the nap session. They were instructed to stay awake, keep the eyes on a fixation point, and quietly listen to auditory streams (2 times 5 minutes of STAT and RDM streams, counterbalanced). No mention was made of regularities.

#### Tone- and tritone-related frequency-tagged responses (whole population)

**Figure 4:**
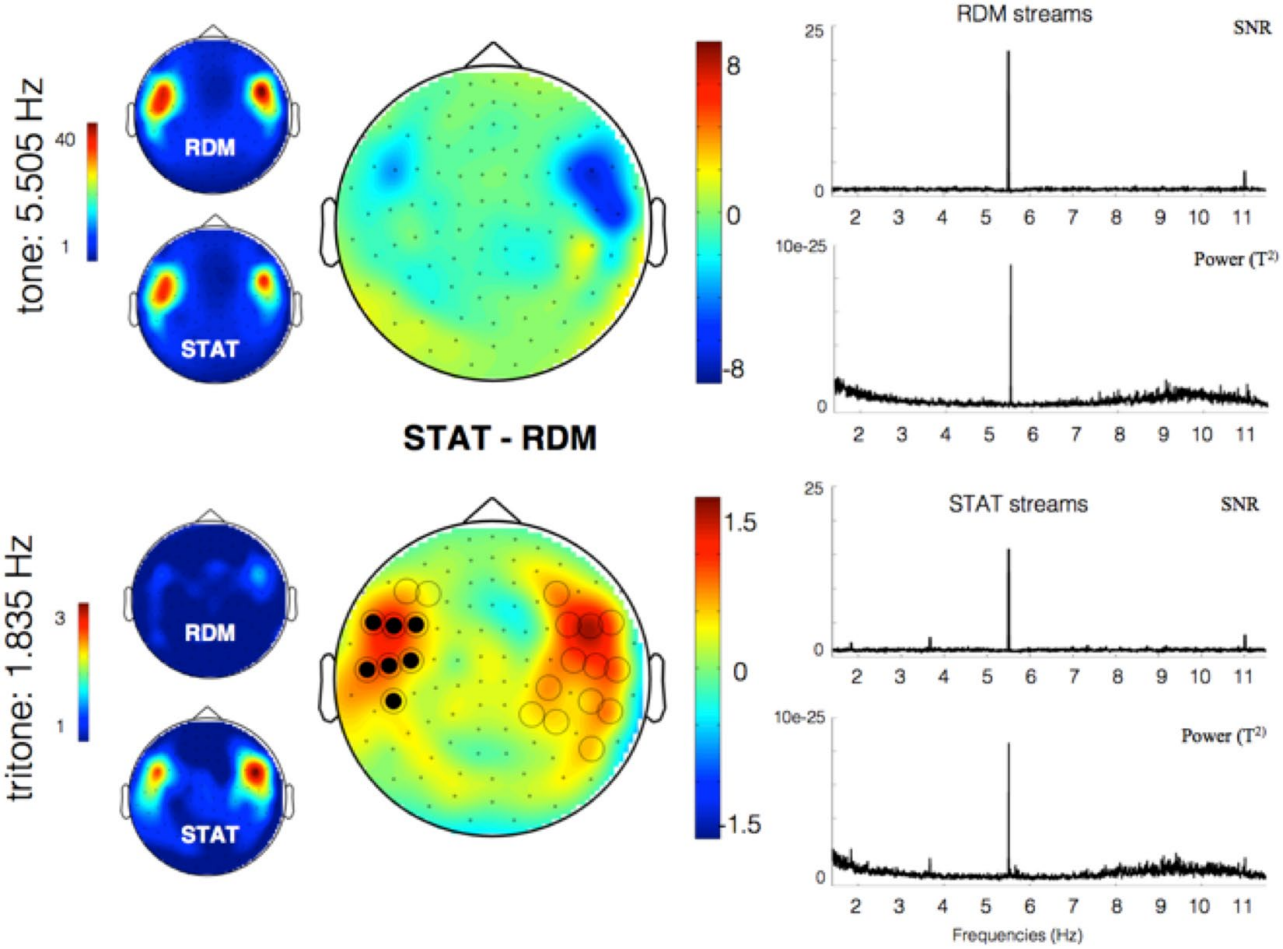
Frequency-tagged responses during the Wake session over the whole population. Topographies, SNR and power spectra (combined gradiometer 1332 + 1333) averaged across participants (n = 21) for tone- (5.505 Hz) and tritone- (1.835 Hz) related frequency-tagged responses for RDM and STAT streams. Left column: Tone-related (up) and tritone-related (bottom) frequency-tagged responses during exposure to STAT and RDM streams. Tritone-frequency tagged response was evidenced for STAT but not RDM streams. Middle column: topographies of differences between STAT and RDM tone- (up) and tritone- (bottom) related frequency-tagged responses. Sensors in significant clusters are marked with filled (p < 0.025) and unfilled (p < 0.05) circles. Right column: SNR and power spectra averaged for RDM (top) and STAT (bottom) streams.

In a first step, we assessed overall segmentation over the entire population (n = 21), pooled, during awake passive listening of STAT and RDM streams, to identify sensors of interest (SOIs). Segmentation corresponded to the averaged 5-min periods for each stream type. Overall power spectra and topographies are illustrated in Fig. 4.

The analysis disclosed a robust tone frequency-tagged response for both STAT and RDM streams. Tone-related SNR was particularly high (SNR > 40) in bilateral temporal sensors. No tritone-frequency tagged response was evidenced during RDM streams. To the contrary, tritone-related SNR for STAT streams was locally increased. A cluster-based permutation test disclosed differences in tritone-related SNR between STAT and RDM streams (p < 0.05) in left and right temporal sensors. SNR and power spectra (Fig. 4, right column) disclosed an emergent peak at the tone-related frequency (and its first harmonic) in both RDM and STAT streams. An emergent peak at the tritone-related frequency (and its first harmonic) was only visible in STAT streams. Sensor space analyses performed on the first harmonic for each frequency of interest (FOI) disclosed similar results (Supplementary Material Fig. S4), reflecting the successful detection of statistical auditory regularities at wake.

#### Tone- and tritone-related frequency-related responses in Sensors of Interest [SOIs] at Wake in NREM/Exposure vs. No NREM/No Exposure participants

**Figure 5:**
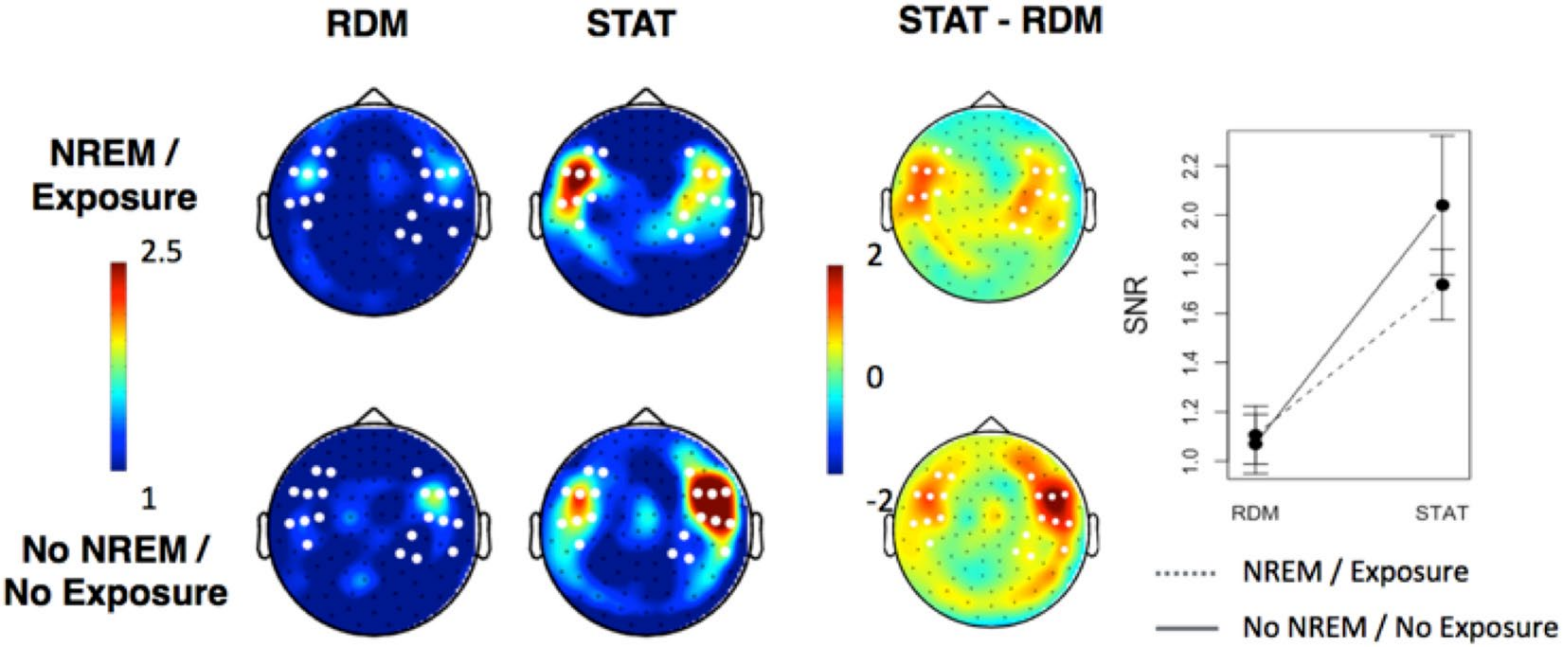
Tritone-related frequency (1.835 Hz) tagging at wake in participants exposed (top) or not (bottom) to the STAT streams in sleep during the prior nap. (Left) Topographies of tritone-related SNR for STAT and RDM (and significant differences STAT - RDM; p < 0.001) in left and right temporal sensors of interests (white dots). (Right) Tritone-related SNR averaged within SOIs for RDM and STAT streams. Group and Group by Stream type interaction effects are non-significant (all ps > 0.4).

Subsequent analyses compared frequency-tagged responses in temporal sensors of interest (SOIs) between participants exposed to the STAT streams during the prior sleep episode (n = 11) and participants without prior exposure due to lack of stable NREM sleep (n = 10). Topographies of tritone-related responses for each group are illustrated Fig. 5.

Tritone-related SNR was averaged within the sensors indicating a global response (i.e., in the clusters identified in the preceding step; see Fig. 4). Mean tritone-related SNR in STAT streams was 1.85 +/− 0.67, greater than baseline value 1 (t(20) = 5.8, p < 0.001). Mean tritone-related SNR in RDM streams was 1.09 +/− 0.38, not different from baseline (t(20) = 1.1, p = 0.3). A repeated measures ANOVA with within-subject factor STREAM (STAT vs. RDM) and between-subjects factor GROUP (Prior Sleep/Exposure vs. Prior Wake/No Exposure) disclosed a main effect of STREAM (F(1,19) = 19.4, p < 0.001), no main GROUP effect (F(1,19) = 0.24, p = 0.4) and no STREAM by GROUP interaction effect (F(1,19) = 0.23, p = 0.6). Separate analyses conducted in left and right clusters disclosed similar results. To probe hemispheric laterality effects, a repeated measures ANOVA with within-subject factor SEQUENCE (STAT vs. RDM) and CLUSTER (LEFT vs. RIGHT) and between-subject GROUP (NREM/no NREM exposure) was conducted. It disclosed again a main effect of the sequence (F = 5.78,p = 0.0016), but no main effect of GROUP or CLUSTER (ps > 0.5), nor any interaction effect (ps > 0.1). Altogether, these results suggest that prior exposure to the STAT stream during sleep did not facilitate the detection of statistical regularities during subsequent wakefulness.

Finally, frequency-tagged responses to regularities were previously shown to develop at wake within the first 3 minutes of exposure^40^. Using linear mixed models, we tested for potential between-group differences in the temporal dynamics of tone- and tritone-related responses, i.e. whether the detection of regularities at wake developed faster in participants previously exposed to the STAT stream during sleep. Results show that the temporal evolution (from first to fifth minute) was not different between participants previously exposed vs. not exposed to the STAT streams during sleep (see Supplementary Material Results Section R1 and Supplementary Material Figs S5-7). Thus, the analyses consistently show that complex stimulus-stimulus associations embedded in statistical regularities remain undetected during sleep, whereas their brain tagging robustly emerges in the same individuals when awake (see individual topographies in Supplementary Material Fig. S2a).

### Behavioural results (2AFC test)

**Figure 6:**
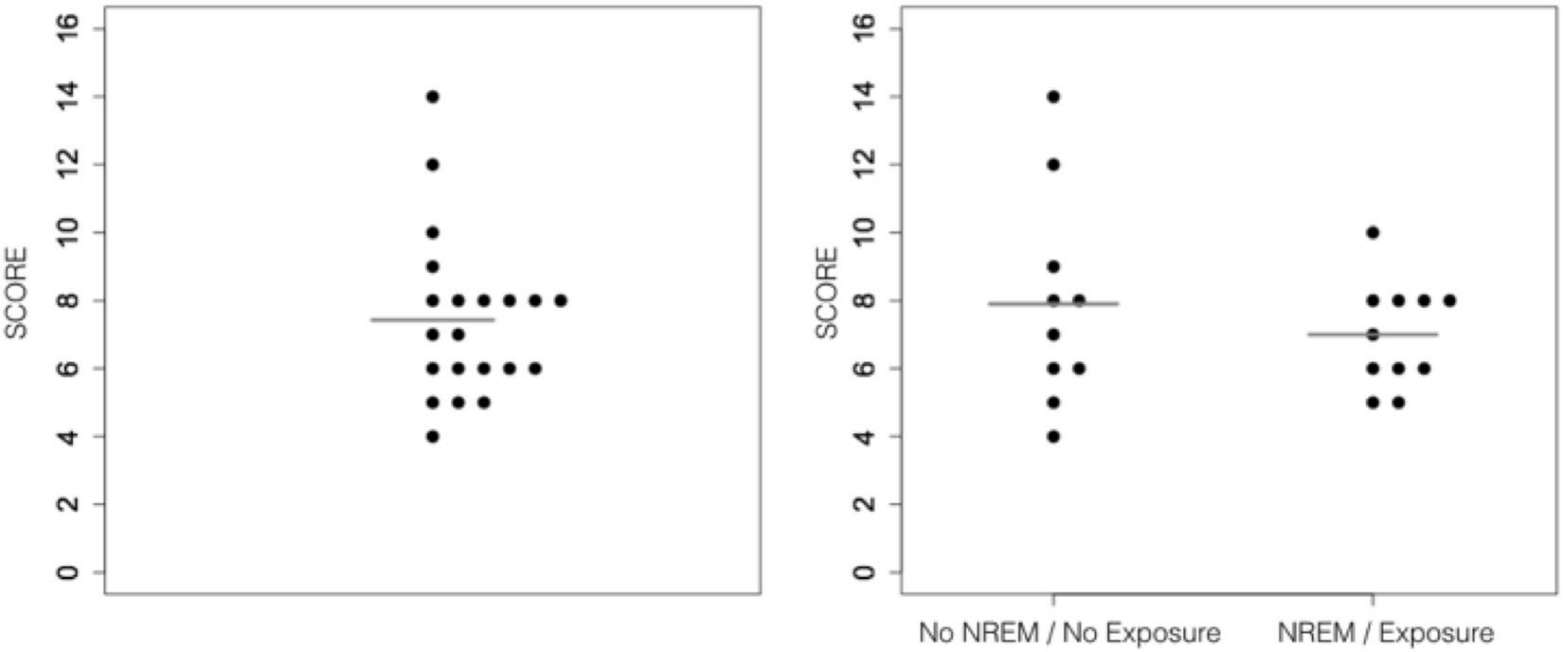
2AFC recognition test. Individual discrimination scores (8/16 = 50% = no discrimination) over the entire population (left) and separately in the NREM/Exposure (Sleep) and No NREM/No Exposure (Wake) participants (right). Horizontal bars indicate average group performance, not different from chance level (p = 0.2).

At the 2AFC test immediately following the Wake (2nd) session, the mean +/− std participants’ recognition performance for both groups pooled was 46.4 +/− 15.0% (range: 25–88%), not different from the 50% chance level (Wilcoxon test, V = 37, p = 0.20). Additionally, we computed a Bayesian unilateral one-sample t-test with chance level as the test value. BF10 was 0.12, providing evidence in favour of the null hypothesis. Mean recognition scores were 49.4 + −19.4% in the No NREM/No Exposure group and 43.8 + −9.7% in the NREM/Exposure group (Fig. 6). Scores were not different between the two groups (Wilcoxon test, W = 61.5, p = 0.67). Lack of between-group differences was substantiated by a Bayesian paired sample t-test in favour of the null hypothesis (BF10 = 0. 24). Participants reported no awareness of the presence of tritones during the stream exposure phase. Additionally, none of the participants reported hearing the streams during sleep.

## Discussion

We took advantage of the power of MEG frequency-tagged responses analyses adapted to the study of statistical learning to investigate whether adults in NREM sleep can detect, and learn, statistical regularities embedded within auditory streams. Frequency-tagged analyses evidenced robust frequency-tagged responses at the tone presentation rate during NREM sleep, but no evidence of segmentation, i.e. no tritone frequency-tagged response during sleep. Nonetheless, all participants successfully segmented the STAT streams during the ensuing wake period, and the temporal evolution of the segmentation process was not different between participants previously exposed vs. not exposed to the STAT streams during NREM sleep. Hence, our results indicate that complex stimulus-stimulus associations embedded in statistical regularities might remain undetected during NREM sleep.

Exposure to auditory streams during NREM sleep elicited robust temporal bilateral responses tagged at the tone presentation rate for both STAT and RDM streams, corroborating studies showing auditory steady-states responses during sleep^48,49^. Tone-related SNR topographies were closely similar to those disclosed at exposure during wakefulness, suggesting that similar neural resources support the auditory processing of tones during sleep and wakefulness, most likely auditory area. There is evidence for similar responsiveness and properties of cortical activity in primary auditory cortices during wake and sleep states^13,16,50–53^. Note that SNR calculations do not allow direct comparisons of amplitude between sleep and wake periods, because background EEG/MEG noise changes with the state^48^. Our use of robust frequency-tagged MEG responses (as compared to ERP experiments) allowed not only group level statistics but also to assess auditory responses at the individual level. All participants, but one, exhibited clear auditory responses at the tone frequency level during NREM sleep both in STAT an RDM streams. Lack of auditory response for the latter participant is unclear. One possibility is that MEG signal was poor (although the same participant elicited clear auditory responses during the wake period). Another possibility would be that this participant had elevated arousal thresholds, precluding correct information transmission to auditory cortices. Indeed, levels of information transfer from thalamus to cortex are influenced by sound intensity in rats^54^, and auditory thresholds may vary from one person to another. Notwithstanding, individual tone-related frequency-tagged auditory responses ensured that deficient auditory processing at lower, primary processing levels was not the cause for lack of learning during sleep.

Despite preserved processing of auditory tones, our analyses failed to evidence segmentation at the tritone frequency of STAT auditory streams during NREM sleep, which would have suggested preserved learning capabilities. More precisely, tritone frequency-tagged responses were at baseline level (SNR = 1) during both STAT and RDM streams. Although a null result could always be accounted for by underpowered samples, or by a too weak SNR in slow frequencies (especially taking into account that during sleep, background EEG/MEG power of low-frequencies <2 Hz is drastically increased, and may contribute to noise levels in our SNR calculations), we are confident that the absence of tritone-related responses genuinely reflects lack of segmentation, for several reasons. First, data inspection shows that noise levels in power spectra were similar between sleep and wake conditions, as can be seen in our figures. Second, we also observed a lack of frequency-tagged responses at the first harmonic (F = 3.67 Hz) of the tritone presentation rate, i.e. outside the NREM3 slow oscillation range. Third, tone-related SNR was not different between STAT and RDM streams. Finally, a high inter-individual variability (which is often the case in implicit learning paradigms) cannot be excluded, with the consequence that some participants learned while others did not. However, once again the high SNR of frequency-tagged responses allowed us to carefully examine responses individually, and in no participant was visible in the spectra a peak at the tritone frequency (whereas tone-related responses were clearly present) even in those exposed up to 20 minutes to the STAT stream. Also, there was no evidence for differences in the evolution of the tritone segmentation process during the ensuing wake period. Altogether, it supports the conclusion that participants were unable to segment streams based on transitional probabilities during their sleep.

Why would statistical learning not be possible during sleep? Learning simple stimulus-response associations^25,26,55^ was already evidenced during NREM sleep in adults, as well as vowels and regularity detection in seemingly sleeping infants^29–31^. In rodents, electrical stimulation of the reward-related medial forebrain bundle during sleep following spontaneous activity in place cells was shown to lead to goal directed behaviours during subsequent wakefulness^56^. Although it suggests that some forms of learning are possible during sleep, other studies reported no traces of memory formation during sleep for more complex learning abilities such as retention of lists of words^3^ or real sounds words^34^. Abolished awareness during sleep seems not a sufficient explanation for lack of higher-order learning, since unconscious learning of more complex forms that conditioning was evidenced during wakefulness, for instance the retention of sequences of symbols unconsciously perceived^57^. Intriguingly, tone-induced conditioned respiratory responses transferrable to wakefulness were evidenced during NREM but not REM sleep^25^, to the opposite of a perceptual noise-memory paradigm in which participants detect repeating noise segments^58^, in which case exposure during REM sleep was beneficial, but exposure during deep NREM3 sleep exerted a detrimental effect on subsequent performance at wake^28^. As we only investigated NREM sleep in the present study, it remains to be ascertained whether statistical learning is as well hindered during REM sleep, or can take place like in this latter study^28^. However, another study investigating high-level speech parsing during sleep found comparable neural tracking of stimulus acoustics across sleep and wakefulness regardless of speech intelligibility, whereas neural tracking of higher-order linguistic constructs such as words or sentences was observed for intelligible speech during wakefulness only, and was not detectable at all during either NREM or REM sleep^59^. As our paradigm similarly involves the high-order detection of statistical regularities embedded within auditory streams, we may expect lack of learning during REM sleep as well, a prediction that remains to be tested.

In this experiment, we provided evidence that statistical learning seems not to develop during NREM sleep. However, statistical learning is a process that occurs at least partially implicitly^60,61^ and learning statistical regularities between syllables was evidenced in REM-like active sleep in newborns^30^. Previous studies evidenced learning during human sleep restricted to perceptual learning^28,29^ or conditioning^25,26^, the latter being subtended by diencephalic and/or subcortical structures (e.g. hippocampus or amygdala). Instead, statistical learning is mostly subtended by medial temporal lobe activity^62^. Although the directionality of the information flow reverses during NREM sleep (from cortex toward hippocampus during wakefulness; to hippocampus toward cortex during NREM sleep^63^), memory-related sound presentation during NREM sleep elicits hippocampal activity in humans^64^ and rodents^65^. Furthermore, trace conditioning (such as tone-odour associations in the Arzi *et al*. study^25^) also relies on hippocampal activity. This makes it unlikely that a disconnection between the hippocampus and sensory areas could account for lack of statistical learning. However, neural structures underlying statistical learning encompass not only medial temporal regions but also a large fronto-parieto-temporal network^66–68^. Reduced metabolic activity in these regions during NREM sleep^69^ might contribute to the difficulty to form novel representations in a statistical learning paradigm during sleep.

In a local-global oddball paradigm, top-down detection of global changes in tones streams was abolished during NREM sleep, whereas bottom-up local changes in auditory stimulation remained partially detected^19^. Similarly, exposure to either pseudo-words or comprehensible sentences during NREM sleep elicited increased activity in thalamus and primary auditory cortex like during wakefulness, but there was no activation of higher-order cortical areas involved in language processing (e.g., Broca’s area)^70^. Still, Wernicke’s area was still activated during NREM sleep exposure in the latter study^70^, but brain responses were similar between pseudo-words and comprehensible sentences, contrary to wakefulness. The authors suggested, in line with Strauss *et al*.^19^, that bottom-up processes are partially preserved during sleep, but that top-down feedback processes are abolished. Makov *et al*.^59^ also showed that alongside preserved low-level auditory processing, higher-level hierarchical linguistic parsing is severely disrupted during sleep. Finally, it was proposed that reduced cortico-cortical connectivity during NREM sleep drastically reduces the brain ability to integrate information^71^. Altogether, deactivation of higher processing brain structures, disrupted top-down processes and reduction of cortico-cortical connectivity during NREM sleep may explain the impaired ability to learn novel high-order statistical regularities.

In the studies mentioned above as well as in the present one, auditory stimulation was delivered regardless of synchronicity with sleep oscillations. Sleep is not a uniform state, and slow waves or sleep spindles are known to largely impact on sound processing. Although it was suggested that sleep spindles originating from the thalamus disrupt auditory perception^72^, rodents data challenged this thalamic gating hypothesis by showing preservation of auditory responses in primary auditory cortices during spindles^73^. Similarly in humans, thalamic and primary auditory activation patterns seem similar during sleep and wakefulness^70^. Still, evidence suggests that auditory processing in higher cortical regions is modulated by the occurrence of sleep spindles, but also by neural bi-stability occurring during NREM sleep, i.e. the fact that sounds are differentially processed during the up and down phase of the slow oscillations^16,34^. These studies indicate that the “gating” may originate more at the cortical than the thalamic level. Such modulations, even if not blocking the primary processing of tones, may impede activity in the higher cortical areas necessary for detection and retention of statistical regularities. Finally, the K-complexes observed in response to sensory stimulation^74^, viewed as mechanisms against cortical arousals^75^, are also known to feature extended cognitive processing of salient stimuli. For instance, activation of the primary auditory cortex increases in response to tones followed vs. not followed by K-complexes^13^. Although a tentative explanation, interactions between spindles, slow oscillations and K-complexes might also prevent/enhance auditory processing in a non-linear fashion, precluding the learning of statistical regularities displayed in a continuous manner.

Finally, another possibility to account for a lack of learning statistical regularities during NREM sleep would be that continuous and rapid succession of stimuli in the auditory streams did not provide sufficient time for plasticity to occur. Indeed, it was shown in TMR studies that tones delivered during a spindle not only result in a disruption of sound perception at higher cortical levels but also disrupts the spindle in itself^73,76,77^. TMR benefits were abolished when two successive sounds were presented at a short interstimulus interval (<1.2 sec), suggesting that a minimal duration is needed to integrate novel information before being able processing the next one during sleep^76,77^. Spindles are also known to promote plasticity in cortical regions^78^, and are related to sleep-dependent memory consolidation processes^79–84^. Interestingly, in the Arzi *et al*.^25^ tone-odour conditioning study during sleep, exposure to tones during learning triggered increases in the spindle related frequency band, again suggesting that spindles play an active role in the encoding of new information. Relatedly, the percentage of trials containing slow frontal spindles correlated with the neurophysiological markers of perceptual learning upon awakening in the Andrillon *et al*. study^28^. It is thus possible that continuous and fast (+/−5 Hz) delivery of tones in the present study disrupted sleep spindles and prevented memory formation.

Although no participant successfully segmented STAT streams during NREM sleep, frequency-tagged responses were significantly increased at the tritone frequency during exposure to STAT (vs. RDM) streams in the Wake condition. Enhanced tritone-related neural responses linearly developed over time in the first continuous five minutes of STAT streams. Topographies of tritone-responses were closely similar to our prior study^40^, with clearly increased responses in bilateral temporal sensors and particularly strong effects in left temporal sensors. Finally, linear increases were also similar (slopes and values) between our two studies. At variance with this prior study^40^ however, tritone responses were no longer present at the second exposure to STAT streams in the Wake session (thus after exposure to RDM streams following the first exposure to STAT streams). It suggests that whereas participants were sensible to statistical regularities during the first exposure, there would be no learning/relearning at second exposure to the STAT streams. One hypothesis to account for this lost effect is that exposure to the RDM stream between the two STAT sequences exerted an interference effect on the learned material and prevented relearning, which would however be in contradiction with our previous report^40^. Dissimilarities between the prior and current experiments might account for different effects. In our first study, participants were allowed a short break at their best convenience after the two first 5-minutes streams. In the present study, the 20 minutes session was continuous. A 20-minutes stimulation sequence may have been too overwhelming for our participants, especially considering the fact that they spent more than 4 hours in the MEG environment scanner, and listened to habituation streams for a large part of the time when not exposed to STAT or RAND streams. In our first study, only 30 minutes were spent in the MEG scanner. Sleepiness, fatigue and decreased attention paid to the auditory stream may thus have played a role. Accordingly, subjective fatigue paradoxically increased in both groups after the nap opportunity (sleepiness being unchanged). Also, participants were lying on their back in supine position, whereas they were seated in the first study, which may have induced position-related increases in sleepiness levels and inattentiveness to the auditory streams. Notwithstanding, expected learning effects were found during the first presentation of the STAT stream. If attention would have decreased of fluctuated with time, then our MEG index should also have progressively decreased/fluctuated. This was not the case as it linearly increased during these first 5 minutes, suggesting that this marker is sensible to learning.

At the behavioural level, performance on the 2AFC task was again at chance level, suggesting that learning of the statistical regularities as evidenced by tritone frequency-tagged responses was implicit. Recent works suggest that learning as assessed by the 2AFC task is largely contaminated by explicit knowledge^42,85^, whereas indirect measures of learning (such as target detection tasks or ERPs measures, and in our case tritone frequency-tagged responses) are more sensitive to implicit learning mechanisms^42^. Altogether, it suggests that tritone frequency-tagged responses during waking exposure reflect implicit learning processes. Regarding tone frequency-tagged responses, although we did not find a significant decrease in the global response at the tone frequency, minute-by-minute analysis showed that tone-response significantly decreased in the STAT (as compared to RDM) streams during the first minutes of the exposure, replicating our initial findings^40^. By contrast, during sleep, no difference was found between exposure to STAT and RDM streams. If the relationship between tritone response and stream segmentation is quite straightforward, interpreting a decrease in tone-related responses is more ambiguous. Several hypotheses can be proposed. First, decreased tone-related SNR in STAT streams may be related to basic (likely bottom-up) processes subtending neural sensitivity to distance lag or repetition (i.e., the time lag that separates two occurrences of the same stimulus^40^). Indeed, time lags are inevitably different between STAT (lag range 6–12) and RDM (lag range 2–12) streams, and sensitivity was previously shown higher at smaller distance lags^86^, possibly enhancing tone-related responses in RDM streams. An alternative but not exclusive possibility would be that diminished tone-related SNR in STAT streams reflects a form of sensitivity to statistical regularities, but not sufficient enough to efficiently perform stream segmentation. For instance, participants could detect that the tone C# is always followed by the tone D, but not segregate tones into tritones. Decreasing tone-related responses would then be related to a suppression-repetition effect driven by top-down expectations^87^. Nonetheless, tone-related response differences between STAT and RDM streams were abolished during NREM sleep. Future studies assessing statistical learning using frequency-tagged responses should investigate in details the mechanisms and implication of tone-related frequency changes.

To sum up, we showed in the present study that in young healthy participants proved able to implicitly learn segmenting auditory streams during wakefulness, segmentation processes seem abolished during NREM sleep. Frequency-tagged responses analysis showed that sleeping participants keep processing auditory information at the tone level, but that auditory processing was not influenced by the presence of statistical regularities in the auditory stream. Therefore, lack of frequency-tagged magnetic responses suggests statistical regularities remained undetected during NREM sleep.

## Methods

### Participants

26 participants (5 males, mean age: 23.7 +/− 2.7 years, range: 19–28) were recruited through public announcement at the Université libre de Bruxelles (ULB, Belgium). The ULB-Hospital Erasme Ethics Committee (Accreditation 021/406) approved the study and all participants gave a written, signed informed consent prior to the experiment. All methods were performed in accordance with the relevant guidelines and regulations for the safe use of magnetoencephalography and electroencephalography. Participants were screened for their facility to sleep in a non-familiar environment, and should not have already been recruited for a similar study. Two participants were excluded due to technical problems during the experiment, 2 for bad EEG signal and 1 due to a lack of tone-related auditory responses during exposure. Descriptions and analyses are restricted to the 21 remaining valid participants. All were in good health with reported normal hearing and no history of neurological or psychiatric disorders, non-musicians, right-handed (Edinburgh Handedness Inventory^88^ mean laterality score +/− sd = 78 + − 20) with normal anxiety levels (State-Trait Anxiety Inventory^89^ French version A mean score +/− sd = 28 +/− 5; version B mean score +/− sd = 38 +/− 7), satisfactory sleep quality (Pittsburgh Sleep Quality Index^90^ global score +/− sd = 3,9 +/− 1,7), and neutral or intermediate chronotype (Morningness-Eveningness Questionnaire^91^ mean score +/− sd = 51,6 +/− 12,1). During the experiment, participants’ sleep quality and quantity for the preceding night were evaluated using the St-Mary’s Hospital sleep questionnaire^92^. They were not sleep-deprived and were asked to keep regular sleep habits the night before, and to avoid energizing drinks the day of the experiment.

Participants were a posteriori assigned to one out of two conditions depending on their level of exposure to the auditory material during the proposed experimental nap. Participants exposed to at least 5 minutes of the statistical (STAT) stream during sleep were assigned to the NREM/Exposure group (N = 11), whereas participants not exposed or exposed less than 5 minutes to the STAT stream were assigned to the No NREM/No Exposure group (N = 10). Chronotype, anxiety, usual sleep quality (PSQI) and laterality levels were not different between groups (Wilcoxon-Mann-Witney test; ps > 0.1).

### Material

Three different types of auditory streams were constructed: statistical (STAT), random (RDM) and habituation (HAB) streams. All streams were composed of twelve pure tones sinus generated with Matlab 2011 (Mathworks Inc., Natick, USA). Tones were all 150 ms duration (5 ms rise and fall) with a 25 ms inter-stimuli interval, recorded at 96 kHz sampling rate, and ranged by half tones from C to B in a single octave in the English musical notation scheme (A = 440 Hz). Stimulus delivery and response collection were controlled using Psychtoolbox-3^93^ running on Matlab 2011 (Mathworks Inc., Natick, USA). Sounds were delivered via a 60 × 60 cm^2^ MEG-compatible high-quality flat panel loudspeaker (Panphonics SSH sound shower, Panphonics, Espoo, Finland).

#### Statistical and random streams

Construction of statistical and random streams is described elsewhere^40^. Only essential information is reported here. Statistical (STAT) streams composed of a set of four possible tritones (G#CD, AC#G, FA#D# and EF#B) were used both in the Nap opportunity and subsequent Wake exposure phases. Tritones were pseudo-randomly determined, with the constraint that no transitional probabilities (TPs) were shared between two sets of tritones, and that tritones did not start nor ended with similar tones. The tritones did not sound melodious with regard to harmonious musical standards to prevent the mental construction of a tune while listening. Within a STAT stream, the four possible tritones were randomly delivered; with the constraint that immediate repetition of the same tritone was proscribed. TPs between individual tones within a tritone and between tritones were thus 100% and 33% respectively (see streams in Fig. 1C, and transition probabilities matrices in Supplementary Material Fig. S8). Random (RDM) streams were composed of the same individual tones concatenated in random order, with the only constraint that a tone was not repeated twice in a row. TPs between tones were thus 9%. In both STAT and RDM streams, an additional blank interval of 20 ms was introduced every three tones (i.e., between tritones in statistical streams) to enhance frequency tagged-responses^39^. Noticeably, this additional interval was shown to be unconsciously perceived^94^, and no frequency-tagged response at the blank interval presentation rate (i.e., identical to the tritones rate) was found in RDM streams in our prior study using the same material^40^, which supports the assumption that these 20 ms blanks are not detected. Stimulus presentation rates were 5.505 Hz for individual tones (i.e., tone duration + inter-stimuli-interval + a third of the subliminal pause) and 1.835 Hz for tritones (i.e., three times smaller than the tone rate). Tone and tritone presentation rates determined the frequencies of interest (FOIs) for the steady-state analysis of the MEG signal (see below).

#### Habituation streams

During the naptime opportunity in the MEG scanner, participants were delivered habituation (HAB) streams when not exposed to STAT or RDM streams. The same twelve tones were continuously played following a natural and expected ascending then descending musical scale succession order (e.g. C/C#/D/D#/E/F/F#…; see Fig. 1C). In 30% of the cases (on average every 12,5 +/− 11,3 seconds, range 2–60 seconds), the ascending scale was repeated, in which case participants had to press a button box. The task is very simple, and participants’ performance was 100% while awake. The purpose of HAB streams was twofold. First, continuous presentations of HAB streams aimed at acclimatizing participants to the acoustic environment and diminish the probabilities of arousal when presenting experimental auditory STAT and RDM streams during sleep. Second, HAB streams aimed at inducing boredom and focusing the participants’ attention toward the tones in a monotonous setting to facilitate sleep onset.

#### Two alternative forced choice (2AFC) task

The 2AFC task aimed at behaviourally probing knowledge of the embedded regularities. It is identical to the one used in our previous study at wake^40^. A concurrent set of four possible tritones (TEST set; DG#F/D#A#A/BC#F#/GCE) was built using the same constraints than for the STAT stream. For each of the 16 trials, two tritones (one from the STAT set and one from the TEST set) were successively presented with a 1-sec interstimulus interval. The 4 tritones of the STAT set were pseudo-randomly combined with the 4 tritones of the TEST set. Hence, each tritone was presented 4 times in a different combination. The order of presentation (first or second within the pair) and the association between tritones from RAND and TEST sets were counterbalanced.

### Experimental procedure

Participants entered the MEG laboratory around 12:30 and started by filling in questionnaires about sleep quality, chronotype, laterality and anxiety (see above), then were prepared for MEG and polysomnography (PSG) recordings (see below).

#### Sleep opportunity session

At about 2:00 pm, participants were installed in the MEG scanner in a confortable supine position, and informed that they would stay in the MEG scanner for about 90 minutes. They were instructed to stay still with the eyes closed while listening to the HAB streams, and to respond by pressing a button box (fORP; Current Designs Inc.) when required (i.e., when there was a repetition of an ascending scale) until they fall asleep. The loudness of the habituation stream was individually adjusted at their best convenience, and they were informed that if auditory streams hindered their sleep, they were allowed to ask diminishing the volume (which was the case for one participant). Ambient lights were either totally switched off or kept at very low intensity at the participant’s convenience. They were not informed that STAT and RDM streams would be displayed during sleep. Polysomnography (EEG, EOG, EMG) was monitored online throughout the 90 minutes period by the main experimenter (JF). When the participant’s exhibited stable and unequivocal NREM2 or NREM3 sleep, presentation of STAT and RDM streams was launched (STAT-RDM-STAT-RDM or RDM-STAT-RDM-STAT, counterbalanced between subjects). Successive intermixed short (5 minutes each) periods of STAT and RDM streams were delivered to minimize possible differences in the evolution of sleep stages between stream types (e.g., to avoid comparison of STAT stream-related neural activity in NREM2 sleep with RAND stream-related neural activity in NREM3 sleep). If a (micro-) arousal or the onset of a REM sleep episode was detected on the polysomnographic online recording, the STAT or RAND stream was immediately replaced by the HAB stream, without transition, then resumed again as soon as stable NREM2/NREM3 sleep conditions were restored. The maximal number of presentations during sleep was 8 × 5-minutes streams (i.e. 4 STAT and 4 RDM streams). If participants were still not sleeping after 60 minutes, they were authorized to leave the MEG room. Before and after the nap opportunity period in the MEG scanner, fatigue and sleepiness levels were assessed using the Visual Analogue Scale of Fatigue and Sleepiness^95^ and the Karolinska Scale of Sleepiness^96^.

#### Wake session

After the nap opportunity period (at about 4:00 pm), participants left the MEG room to fully wake up and dissipate sleep inertia^97^. After maximum 30 minutes, they were once again installed in the MEG scanner in supine position, with the difference that they were required to keep the eyes open and stay awake, with the ambient lights on. They were then presented 4 × 5 minutes of STAT/RAND streams (STAT-RDM-STAT-RDM or RDM-STAT-RDM-STAT, counterbalanced). They were instructed to quietly listen to the auditory streams while fixating a fixation cross on the ceiling, but did not receive any explicit instructions to detect regularities.

After this second exposure session, participants were informed about the presence of regularities and presented the 2AFC test (see above). Pairs of tritones (n = 16) were aurally displayed, and participants had to indicate aloud which one of the two tritones was part of the exposure streams, either because they recognized it or because it sounded most familiar. The 2AFC test lasted around 5 minutes. Due to the small number of trials, MEG data were not recorded during this recognition task.

### Polysomnography (PSG) and MEG data acquisition

#### Polysomnography (PSG)

PSG was acquired to determine (on-line and off-line) sleep stages. The EEG setting included 3 derivations (C3-A2, C4-A1 and Fz-A1) positioned according to the 10–20 electrodes placement system^47^. MEG-compatible single gold-plated electrodes (Reference E6650 IMMED, Belgium) were secured on the scalp using collodion, and impedance was kept below 10 Kohm. Bipolar EOG (recording both vertical and horizontal ocular movements), bipolar EMG on the chin and bipolar ECG were additionally recorded using similar electrodes. All electrodes were directly connected to the MEG setting to ensure synchronization between EEG and MEG signals. Online monitoring allowed delivering auditory streams in correct (NREM2 or deeper) sleep conditions. Accuracy of online detection was verified, and additional analyses conducted offline on EEG and EMG data band-pass filtered at 0,30–30 Hz and 10–100 Hz respectively, and sleep staging performed based on 30-s epoch windows according to the AASM criteria^47^.

#### Magnetoencephalography (MEG)

MEG data were recorded using a whole-scalp-covering 306-channel neuromagnetometer (102 sensor chipsets, each comprising one magnetometer and two orthogonal planar gradiometers) installed in a light-weight magnetically shielded room (Vectorview and MaxShield; Elekta Oy, Helsinki, Finland), the characteristics of which are described elsewhere^98^. The MEG signal was recorded at a sampling rate of 1 kHz using a band-pass filter set at 0.1–330 Hz. Head position was continuously monitored using four head-tracking coils. The locations of the coils and at least 150 head-surface (on scalp, nose, and face) points with respect to anatomical fiducials were digitized using an electromagnetic tracker (Fastrack, Polhemus, Colchester, VT).

To ensure accurate synchronization for frequency-tagged analyses (see below), a trigger was sent in the MEG recording every 3 tones via a parallel port Arduino system (https://www.arduino.cc). Triggers were also sent into the MEG recording when there was a repetition during HAB streams and when participants pressed the button box.

### Data analysis

#### MEG data pre-processing

External interferences and head movements were corrected offline using the signal space separation (SSS) method^99^. In addition, MEG data of each participant were realigned using the SSS method on the first MEG run of the first participant, to align the recordings of all subjects into a common sensor space.

#### Frequency-tagged analyses

All reported analyses were conducted in the sensor space using Matlab R2011a and Fieldtrip^100^. Separate but identical analyses of MEG data were conducted during the Sleep opportunity session for participants exposed to at least 5 min of the STAT stream (NREM/Exposure group; N = 11), and during the subsequent Wake sessions for both the NREM/Exposure (N = 11) and the No NREM/No Exposure (N = 10) groups.

Overall exposure to STAT and RDM streams: In a first step, frequency-tagged analyses aimed at evidencing global segmentation-related responses during exposure to RDM (tone-related) and STAT (tone- and tritone-related) streams. These analyses we conducted only in participants continuously exposed to at least 5 minutes (i.e., 560 cycles of the tritone frequency, skipping the first 12 tones to avoid transient effects) in both STAT and RDM streams. In the Wake (second) session, all participants were by design exposed to 4 × 5 minutes of STAT/RDM streams. In the Sleep nap opportunity (first) session, the duration of exposure to STAT and RDM streams varied from one participant to the other (see Supplementary Material Fig. S1 and Table T1) in the Prior Sleep/Exposure group, with two participants exposed to 5 minutes of STAT but not of RDM streams.

In each stream condition, the 5-min data were averaged to increase signal to noise ratio (SNR), thus dampening signal not phase-locked with auditory stimulus delivery. Computations on long 5-min epoch (frequency resolution 0.0033 Hz) ensure a high spectral resolution. MEG power spectra were computed using a Hanning taper Fast Fourier Transform (FFT) in the 1.5–12 Hz range. SNR of spectra power was computed for each frequency bin as the ratio between the power values of the bin and the averaged power of the 200 neighbouring frequency bins (skipping the two closest neighbouring frequency bins), calculated on combined gradiometers (i.e. the sum of power frequencies of both radial orientations). Tone-related SNR responses during sleep were visually inspected for each participant separately (individual topographies are available in Supplementary Material Fig. S2). Grand average tritone- and tone-related SNR across all participants were calculated for each condition (STAT and RDM streams) in each session.

Statistical analyses were focused on the two frequencies of interest (FOI; i.e., the tritone presentation rate at 1.835 Hz and the tone rate at 5.505 Hz), separately for the Sleep nap opportunity and the Wake sessions. To assess differences in tone- and tritone-related SNR between RDM and STAT streams, statistical analyses were conducted using non-parametric Monte-Carlo estimates from the permutation method (10′000 permutations, paired comparisons, computed for two levels of significance: alpha = 0.05 and alpha = 0.025, unilateral) and the cluster based statistics (alpha cluster = 0.05 and 0.025, unilateral) to control for multiple comparisons problem as implemented in Fieldtrip^100^. Cluster based analyses were performed using the neighbours definition template based on the triangulation method for Neuromag systems as provided by Fieldtrip (see template in Supplementary Material Fig. 9). Similar analyses were also conducted on the first harmonics of each FOIs (see Supplementary Material Figs S3 and S4).

Temporal evolution of tone and tritone frequency-tagged responses: In a second step, frequency-tagged analyses aimed at evidencing the temporal evolution of segmentation-related responses during the 5 minutes of exposure to the RDM and the STAT streams, as a function of the experimental group (NREM/Exposure vs. No NREM/No Exposure). For this purpose, MEG data were cropped into 1-min time windows (i.e. 61.04 seconds containing 112 cycles of the tritone frequency). FFT analyses similar to those described above were applied to each 1-min block (frequency resolution 0.0164 Hz). Fourier spectra values were successively averaged over two minutes to increase statistical power (i.e. minutes 1–2, 2–3, 3–4 and 4–5). Finally, FFT power and SNR were calculated as the ratio between power at a frequency and its 40 neighbouring frequencies (skipping the two closest).

Sensors of interest: We also determined sensors of interest (SOIs) for subsequent linear mixed models analyses (see next subsection). In the Wake (2nd) session, SOIs for tritone-related responses were selected as sensors in which tritone-related SNR was significantly above the value 1 (10′000 permutations, alpha = 0.05, uncorrected) at the end of the two 5-min STAT streams. Actually, those sensors also corresponded to all but one with a SNR > 1.25, i.e. a minimum of a 25% power increase as compared to neighbouring frequencies. For the tone-related responses, almost all sensors had SNR > 1. Thus, we selected SOIs as sensors in which averaged tone-SNR during minutes 1–2 of RDM streams was >5 (i.e. tone frequency power 5 times greater than in neighbouring frequencies). SNR values were averaged across SOIs for each FOI. In the Sleep nap opportunity (first) session, no sensor exhibited tritone-SNR > 1 at the end of STAT streams (ps > 0.05, uncorrected). Therefore SOIs were selected as those identified during the subsequent Wake period. For the tone-related responses, SOIs were selected as sensors in which SNR was >2 during minutes 1–2 of RDM streams.

Linear mixed models: Linear mixed models (LMM) were used to model the temporal dynamics of the tone and tritone responses in SOIs, reflecting the gradual evolution of tone detection and segmentation (tritone detection) processes during the two sessions. We used the “lme4” and “lmerTest” packages in the R environment (https://www.r-project.org/). In the present study, LMM featured several advantages as compared to repeated measures ANOVA or linear regressions. First, LMM deal with variant covariance between the different levels of temporal evolution across successive recorded minutes within a stream (i.e. factor MINUTES). Indeed, it is typical in learning paradigms (and even more especially in the present case since time slots were averaged) that correlation is different between data collected at different time points (for instance, tritone-SNR in statistical streams during the Wake session was more correlated between minutes 2/3 and 3/4 [R = 0.6, p < 0.001] than between minutes 2/3 and 4/5 [R = 0.2, p = 0.2]). Similarly, LMM deals with unequal variance between different levels in the factor MINUTES. Again, bigger SNR variance was expected (and observed) at the end of the exposure phase than at the beginning. Finally, LMM provides indications about the relationships between dependent measures and predictors (i.e., it indicates whether the relationship is categorical, linear, quadratic,…).

Sleep nap opportunity session: In the NREM/Exposure group, the first continuous 5-min RDM and STAT streams were selected for each participant. Model building was performed in two steps and separately for both tone- and tritone- responses. Fixed effects were the predictors MINUTES (1–2 vs. 2–3 vs. 3–4 vs. 4–5; categorical, continuous or quadratic) and STREAM type (STAT vs. RDM).

In the first step, the most appropriate model of the covariance structure of the data was selected. To do so, we defined all the possible combinations of the random effects accounting for the data (i.e., we specified random intercepts representing the correlation within participant, and a random slope of the MINUTE and STREAM predictors to account for the variation between participants in the effects). We used the most saturated model for the fixed effects (i.e., containing all the categorical predictors MINUTES and STREAM and the corresponding interactions) and the Residual Maximum Likelihood Method (REML), while varying the structure of the covariance (i.e., the specific combination of random effects). The most appropriate model for the covariance structure was selected based on the Akaike Information Criterion (AIC) corrected for small sample sizes. The AIC is a model selection criterion typically used for the covariance model selection (and also fixed effect model selection) when the different models compared are not nested. This criterion measures the relative quality of statistical models for a given data set: the smaller the AIC, the more parsimonious and appropriate is the model.

In the second step of model building, using the selected covariance structure identified in the first step and the Maximum Likelihood (ML) method, we selected the most appropriate model for the fixed effects. We derived all the possible fixed effect models, i.e., all the possible combinations of predictors STREAM and the categorical, continuous or quadratic predictor MINUTES. The model that best accounted for the data was chosen based on the AIC. Therefore, predictors and/or interactions terms not included in the final model did not add information that could improve model fitting.

Wake session: In the subsequent Wake session, model building was similarly performed in two steps and separately for the first and second five minutes streams for both tone- and tritone- responses. The between-subjects predictor GROUP (NREM/Exposure vs. No NREM/No Exposure) was added to the predictors MINUTES (1/2 to 4/5) and STREAM (STAT vs. RDM streams), and accounted for in all combinations as explained above. Incorporation of the GROUP factor aimed at determining whether exposure to the STAT auditory streams during the prior sleep opportunity in the NREM/Exposure group led to a faster detection/segmentation of the tritones during the Wake session, as compared to the No NREM/No Exposure group, which would indicate that subjects exposed to STAT streams during their sleep were sensitized to their underlying tritone structure.

### Behavioural analyses

Statistical analyses on behavioural performance at the 2AFC test were conducted using R package (https://www.r-project.org/). A correct response was defined as the accurate recognition of a tritone being part of the exposed STAT stream. Total scores in percentage (/16*100) were assessed for each participant. Since values were not normally distributed, performances of participants from both the No NREM/No Exposure and the NREM/Exposure groups were first pooled together and performance tested using the non-parametric Wilcoxon rank sum test against chance level (50%). In a second step, between-group differences were assessed using an independent Wilcoxon test between scores.

### Bayesian analyses

Bayesian analyses were computed where needed to provide additional characterization in the context of statistically null results. Bayesian analyses were computed using the free software JASP (Version 0.9) with default priors (JASP Team, 2018). By convention, a Bayes Factor (BF) > 3 is considered as substantial evidence for the alternative hypothesis (H1), BF values < 0.333 indicate substantial evidence for the null (H0), and BF values between 0.333 and 3 are inconclusive^101^.

### Data Availability

The datasets generated during and/or analyzed during the current study are available from the corresponding author on reasonable request.

## Electronic supplementary material

Supplementary Material
