# Supplementary material for: Lack of frequency-tagged magnetic responses suggests statistical regularities remain undetected during NREM sleep
Source: Sci Rep. 2018 Aug 6;8:11719. doi: 10.1038/s41598-018-30105-5 (PMC6079006; doi:10.1038/s41598-018-30105-5)
Supplement: Supplementary file 1 — Supplementary Material [file 41598_2018_30105_MOESM1_ESM.pdf]

Lack of frequency-tagged magnetic responses suggests statistical regularities remain undetected during NREM sleep

FARTHOUAT Juliane, ATAS Anne, WENS Vincent, DE TIÈGE Xavier, and PEIGNEUX Philippe

SUPPLEMENTARY MATERIAL

Supplementary Material Figure S1

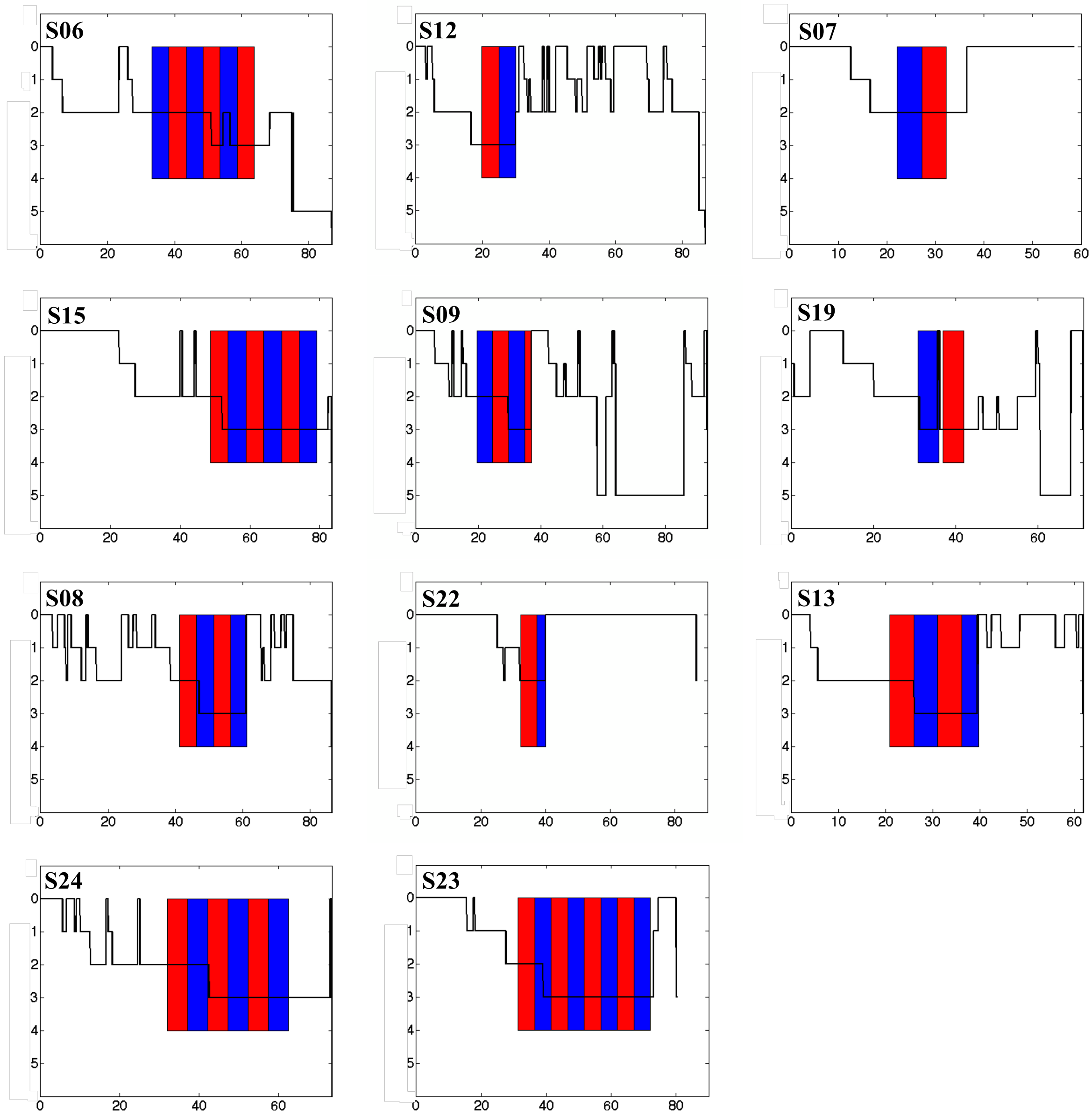

S1. Individual hypnograms (sleep profile) for the 11 participants in the NREM / Exposure group. Red bars: exposure to STAT streams. Blue bars: exposure to RDM streams. Wake, NREM1, NREM2, NREM3 and REM sleep are coded 0, 1, 2, 3 and 5 (y axis) respectively. Time (x axis) is expressed in minutes.

Supplementary Material Figure S2a

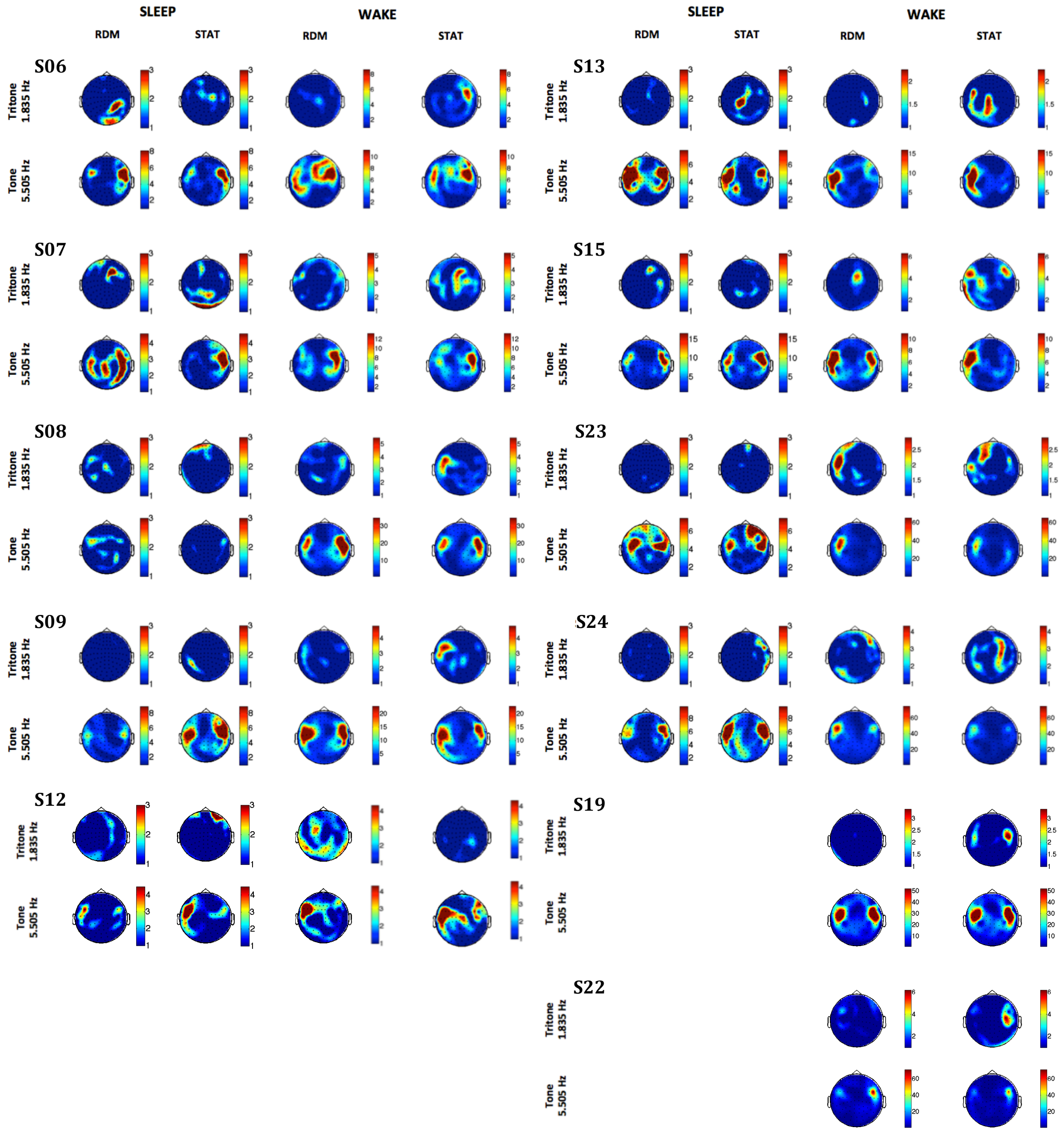

S2a. NREM / Exposure group. Individual topographies for tone- and tritone-related frequency-tagged responses in STAT an RDM conditions in the Sleep nap opportunity and subsequent Wake sessions. Note that participants S19 and S22 were exposed to 5 minutes of STAT, but less than 5 minutes of RDM streams during the sleep opportunity (see Supplementary Material Figure S1 and Table T1), and were thus excluded from statistical analyses (contrast STAT vs. RDM) for this period. SNR amplitude colour codes are not homogenized between conditions.

Supplementary Material Figure S2b

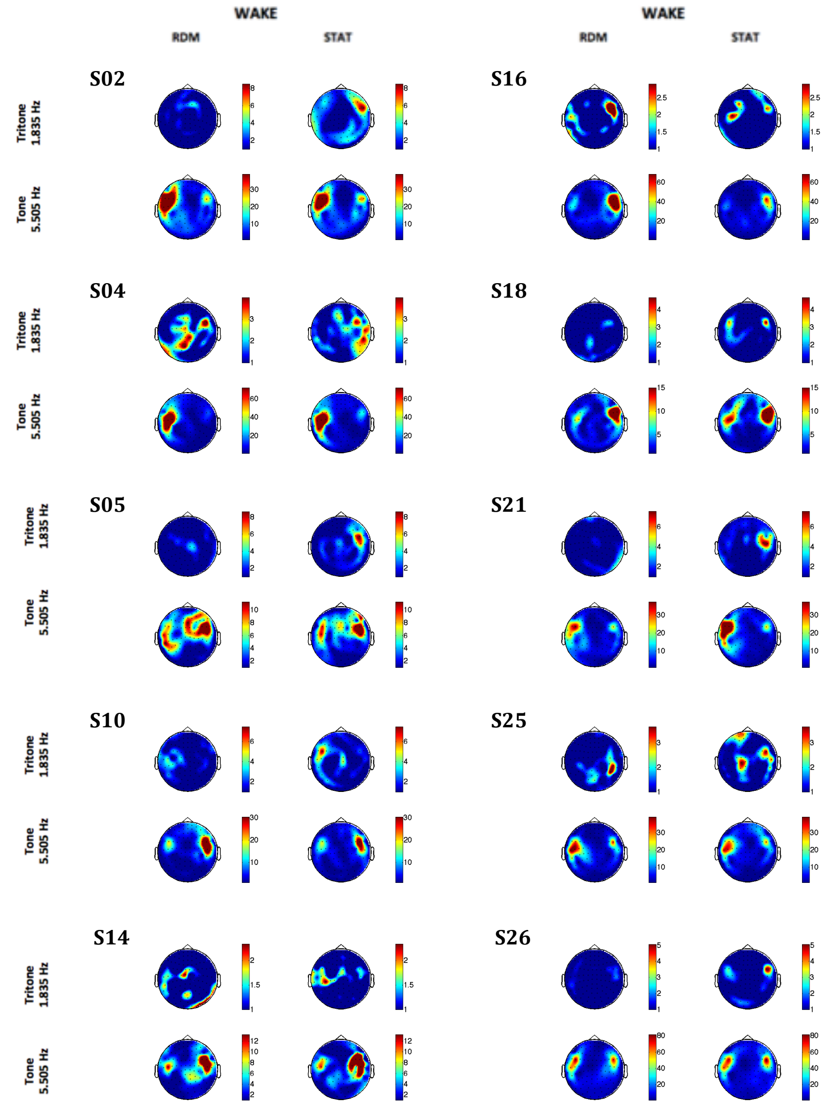

S2b. No NREM / No Exposure group. Individual topographies for tone- and tritone-related frequency-tagged responses in STAT an RDM conditions in the Wake (2nd) session. SNR amplitude colour codes are not homogenized between conditions.

Supplementary Material Figure S3

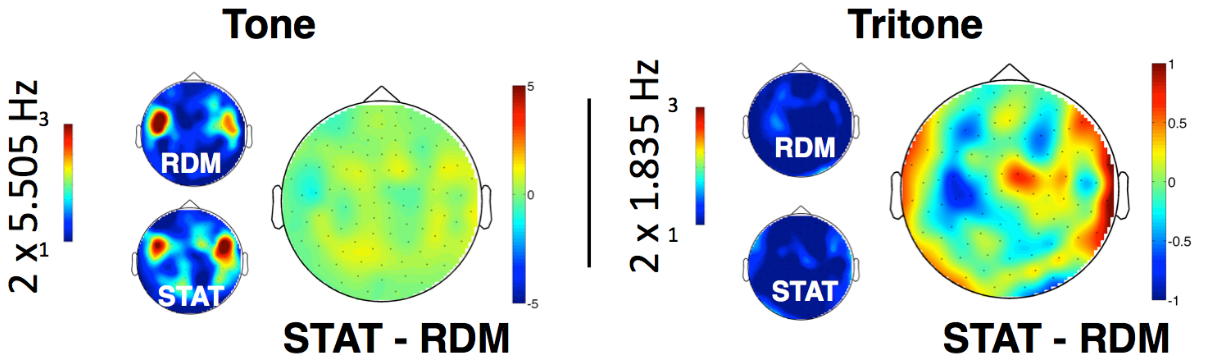

S3. Sensor space analyses performed at the first harmonic of each frequency of interest (FOI) during sleep in the NREM / Exposure condition. Topographies averaged across participants for the first harmonic of the tone- (left panel) and tritone- (right panel) related frequency tagged responses during NREM sleep. There is no difference between statistical (STAT) and random (RDM) streams for both tone- and tritone-related responses (see main text).

Supplementary Material Figure S4

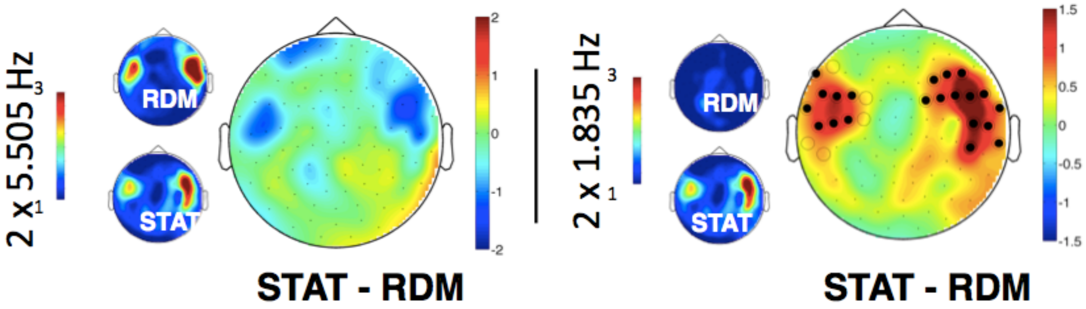

S4. Sensor space analyses performed at the first harmonic of each frequency of interest (FOI) during the Wake session (all participants). Topographies are averaged across all participants for the first harmonic of the tone- (left panel) and tritone- (right panel) related frequency tagged responses during NREM sleep. There is no difference between statistical (STAT) and random (RDM) streams for tone-related responses (see main text).

SUPPLEMENTARY MATERIAL RESULTS SECTION R1

Time course of tone- and tritone-related responses at Wake in NREM / Exposure vs. No NREM / No Exposure participants

Potential between-group differences (NREM / Exposure vs.No NREM /No Exposure) in the temporal dynamics of tone- and tritone-related responses were assessed using linear mixed models (LMM).

1. Dynamics of tritone-related frequency-tagged responses

The time course of the tritone-related responses averaged within sensors of interest (including left and right temporal sensors) is illustrated in Supplementary Material Figure S5. Time courses of tritone-related SNR were examined separately for the first and the second 5-minutes streams.

For the first 5-minutes STAT and RDM streams, two models had the same smallest AIC value. These models included the main and interaction terms between continuous MINUTES and STREAM predictors for both linear and quadratic MINUTES terms (i.e. SNR ~ STREAM*MINUTES and SNR ~ STREAM*MINUTES^2, AIC=329.9). This indicates that the predictor GROUP did not add information that could improve model fitting. Because the linear model was the one with the fewest parameters, we selected this model as the most appropriate one. A significant linear increase of the tritone-related SNR response with time (i.e., across successive minutes) was observed in STAT streams (estimate of the MINUTES slope = 0.17 +/- 0.06, $t(51.54) = 2.7$, $p<0.01$; intercept estimate =0.88 +/- 0.15) but not in RDM streams (estimate of the MINUTES slope = 0.04 +/- 0.06, $t(51.54) = 0.6$, $p=0.5$; intercept estimate =0.92 +/- 0.17). Individual slopes (see Supplementary Material Figure S6) for STAT streams were positive for all but one participant. Intercepts of the MINUTES regression line were similar between STAT and RDM streams (estimate = -0.041 +/- 0.23, $t(108.87) = -0.17$, $p=0.9$). Finally, the interaction between continuous MINUTES and STREAM was marginally significant (the estimate of the slope difference between random and statistical was 0.14 +/- 0.07, $t(126)= 1.8$, $p=0.065$). To sum up, tritone-related SNR during STAT streams started at baseline level (i.e., SNR ~ 1) and linearly developed up to a ~50% increase at the end of the 5-min STREAM. In contrast, tritone-related SNR during RDM streams remained at baseline level throughout exposure.

Supplementary Material Figure S5

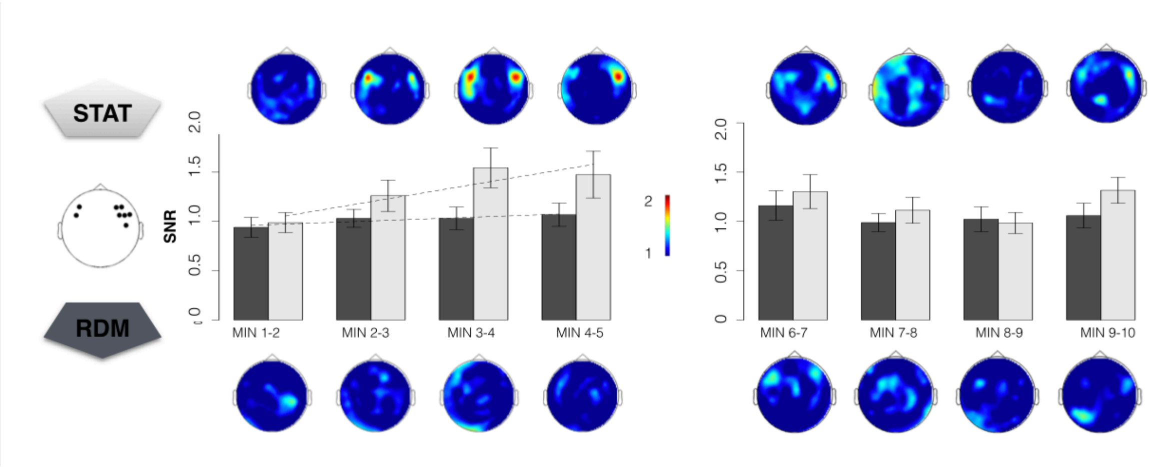

S5. Temporal dynamics of tritone-related SNR for the first (left) and second (right) 5-minutes exposure during the Wake (2nd) session. Data are pooled over NREM / Exposure and No NREM / No Exposure participants as the Group effect was non significant. Topographies are related to STAT (top) and RDM bottom) streams. Averaged tritone-related SNR within sensors of interest (SOI) are displayed in light grey bars for STAT streams and dark grey bars for RDM streams. During the first 5-minutes streams, tritone-related SNR linearly increased in STAT streams while remaining at baseline level (~1) in RDM streams. During the second 5-minutes STREAMs, no particular pattern emerged. Error bars indicate standard error. Dashed lines indicate linear regressions estimated from mixed models analyses.

For the second five minutes STAT and RDM streams, the most appropriate model included the categorical MINUTES, STREAM and GROUP predictors but no interaction term (i.e. SNR ~ MINUTES + STREAM + GROUP). Tritone-related SNR tended to decrease during minutes 2-3 (estimate of the difference=-0.18 +/- 0.11, t(133.8)=-1.7, p=0.09) and 3-4 (estimate of the difference =-0.23 +/- 0.12, t(57.4)=-1.8, p=0.06), as compared to the minutes 1-2, but not during minutes 4-5 (estimate of the difference =-0.044 +/- 0.14, t(24.97)=-0.3, p=0.7). Importantly for our purpose, tritone-related SNR did not differ between STAT and RDM streams (estimate of the difference =0.12 +/- 0.10, t(21.7)=1.2, p=0.2) and exhibited a trend to be higher in the Prior Sleep/Exposure than in the Prior Wake/No Exposure group (estimate of the difference =0.21 +/- 0.12, t(23.5)=1.7, p=0.09).

Supplementary Material Figure S6

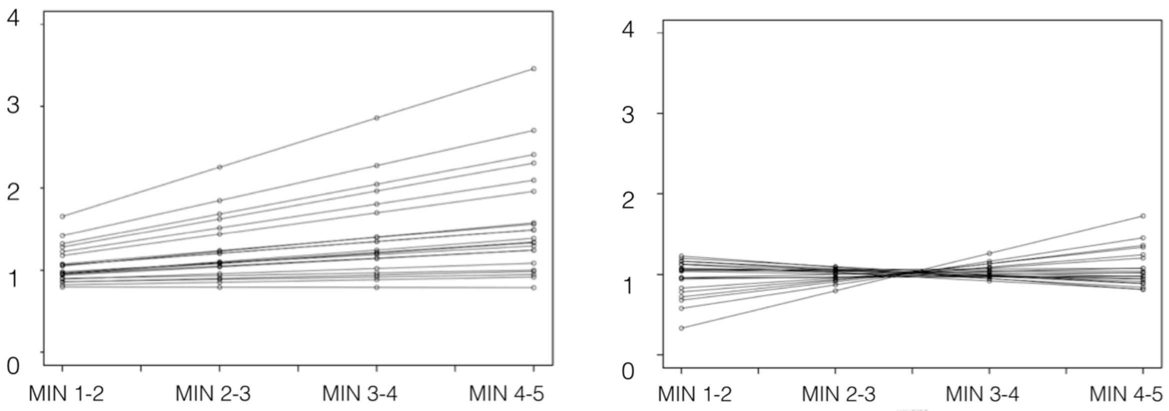

S6. Individually fitted random slopes and intercepts (SNR) for the first 5-min STAT (left) and RDM streams (right).

2. Dynamics of tone-related frequency-tagged responses

Similarly, the time course of the tone-related responses averaged within sensors of interest (left and right temporal sensors) is illustrated in Supplementary Material Figure S7, and time courses of tone-related SNR were examined separately for the first and the second 5-minutes STREAMs. Based on the AIC, random intercepts and random slopes for MINUTES and STREAM were included in the model.

For the first five minutes streams, the most appropriate models for the fixed effect included the three predictors and interaction terms between continuous MINUTES and STREAM predictors for both linear and quadratic MINUTES terms (i.e. SNR ~ STREAM*MINUTES and SNR ~ STREAM*MINUTES^2, AIC=765.4). The tone-related SNR responses linearly decreased across minutes for RDM streams (estimate of the minute slope = -0.6 +/- 0.2, t(37.82)= -3.1, p<0.01, intercept estimate=8.0 +/- 0.9) but not for STAT streams (estimate of the minute slope= -0.00 +/- 0.2, t(37.82)=0.0, p=0.99; intercept estimate =5.4 +/- 0.9). Intercepts of the MINUTES regression line between STAT and RDM streams were significantly different (estimate of the difference in intercept = 2.6 +/- 0.9, t(37.12)=2.7, p=0.01). The interaction between MINUTES and STREAM was also significant (estimate of the slope difference between RDM and STAT = 0.6 +/- 0.2, t(105)= -3.0, p=0.003). Hence, at the beginning of auditory streams exposure, tone-related SNR for STAT streams was lower than for RDM streams, but this difference was no longer significant at the end of the streams. In addition, tone-related SNR in RDM streams linearly decreased as a function of time.

Supplementary Material Figure S7

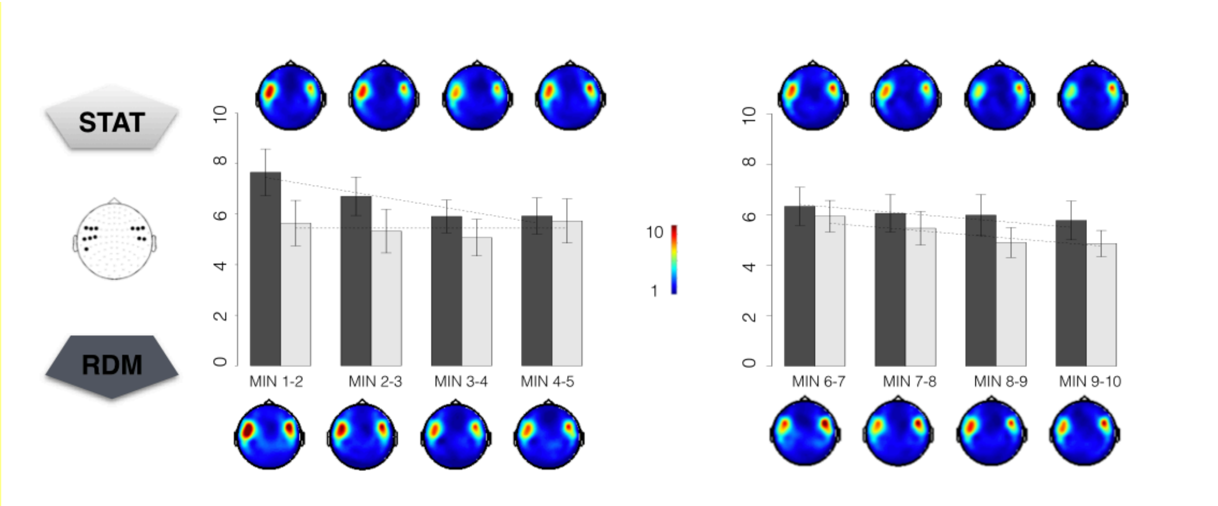

S7. Temporal dynamics of tone-related SNR for the first (left) and second (right) 5-minutes exposure during the Wake (2nd) session. Data are pooled over NREM / Exposure and No NREM / No Exposure participants as no Group effect was evidenced. Topographies are related to STAT (top) and RDM bottom) streams. Averaged tone-related SNR within sensors of interest (SOI) are displayed in light grey bars for STAT streams and dark grey bars for RDM streams. During the first 5-minutes STREAMs, tone-related responses linearly decreased in RDM streams, whereas it remained constant in STAT streams. During the second 5-minutes STREAMs, tone-related SNR similarly decreased in RDM and STAT streams. Error bars indicate standard error. Dashed lines indicate linear regressions estimated from mixed models analyses.

For the second 5-min streams, the most appropriate model for the fixed effect included predictors STREAM and continuous MINUTES (i.e., SNR ~ STREAM + MINUTES). Tone-related SNR decreased across minutes both for STAT (slope estimate =-0.23 +/- 0.11, t(21)=2.4, p=0.03, intercept estimate = 6.0 +/- 0.7) and RDM (slopes estimate=-0.28 +/- 0.11, t(21)=-2.4, p=0.03, intercept estimate = 6.7 +/- 0.8) streams. There was no difference in intercept between streams (estimate of the difference=0.75 +/- 0.44, t(21)=1.7, p=0.1). In other words, tone-response did not differ between STAT and RDM streams, and linearly decreased over time.

Supplementary Material Figure S8

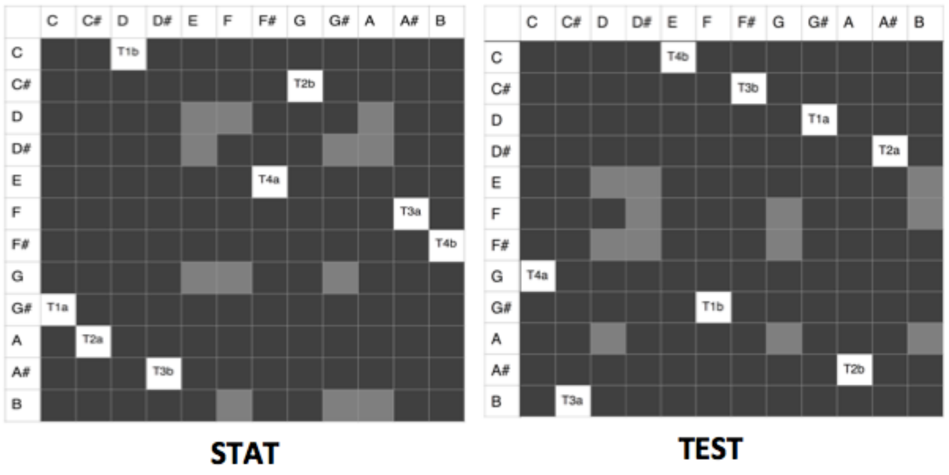

S8. Transition probabilities matrices in statistical streams. The STAT set is used for exposure during the Nap opportunity and the subsequent Wake sessions, the TEST set is additionally used in the behavioural discrimination (2AFC) test (see 2AFC section below). Black, grey and white boxes respectively indicate transitional probabilities of 0, 33 and 100%. STAT and TEST sets share no positive transitional probabilities. None of the tritones from STAT and TEST sets started or ended with the same tones.

Supplementary Material Figure S9

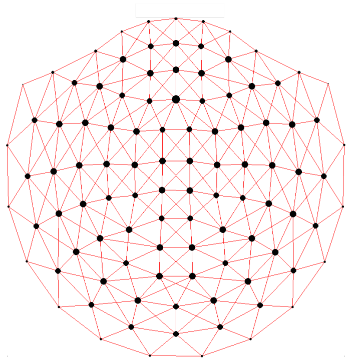

S9. Neighbours definition template based on the triangulation method for Neuromag systems provided by Fieldtrip (Oostenveld R, Fries P, Maris E, & Schoffelen JM FieldTrip: Open source software for advanced analysis of MEG, EEG, and invasive electrophysiological data. Comput Intell Neurosci, 2011, 156869. doi:10.1155/2011/156869)

Supplementary Material Table T1

		RDM	STAT	Total
**S06**	**S2**	12.5	7.5	20.0
	**S3**	2.5	5.5	8.0
**S07**	**S2**	5.0	5.0	10.0
	**S3**	0.0	0.0	0.0
**S08**	**S2**	0.0	5.0	5.0
	**S3**	10.0	5.0	15.0
**S09**	**S2**	5.0	5.4	10.4
	**S3**	5.0	0.6	5.6
**S12**	**S2**	0.0	0.0	0.0
	**S3**	5.0	5.0	10.0
**S13**	**S2**	5.0	5.0	10.0
	**S3**	5.0	2.5	7.5
**S15**	**S2**	0.0	3.1	3.1
	**S3**	15.0	11.9	26.9
**S19**	**S2**	0.0	0.0	0.0
	**S3**	4.5	5.0	9.5
**S22**	**S2**	2.5	5.0	7.5
	**S3**	0.0	0.0	0.0
**S23**	**S2**	2.5	5.0	7.5
	**S3**	17.5	15.0	32.5
**S24**	**S2**	5.0	5.0	10.0
	**S3**	10.0	10.0	20.0

T1. Duration of exposure (minutes) to STAT and RAND streams, per participant and per sleep stage in the NREM / Exposure group. S2 = NREM2 Sleep; S3 = NREM3 Sleep

Supplementary Material Table T2

	RDM	STAT
**S02**	0	0
**S04**	4 min 7 sec	0
**S05**	0	0
**S10**	5 min	2 min 16 sec
**S14**	0	1 min 47 sec
**S16**	0	26 sec
**S18**	0	1 min 13 sec
**S21**	0	0
**S25**	0	0
**S26**	0	0

T2. Duration of exposure to RAND and STAT streams for each participant in the No NREM / No Exposure group (i.e., with less than 5 minutes exposure to STAT streams)
